# Functional and genomic analyses reveal therapeutic potential of targeting β-catenin/CBP activity in head and neck cancer

**DOI:** 10.1186/s13073-018-0569-7

**Published:** 2018-07-20

**Authors:** Vinay K. Kartha, Khalid A. Abalkhail, Khikmet Sadykov, Bach-Cuc Nguyen, Fabrice Laroche, Hui Feng, Jina Lee, Sara I. Pai, Xaralabos Varelas, Ann Marie Egloff, Jennifer E. Snyder-Cappione, Anna C. Belkina, Manish V. Bais, Stefano Monti, Maria A. Kukuruzinska

**Affiliations:** 1 Bioinformatics Program, Boston University, Boston, MA USA; 2 Division of Computational Biomedicine, Boston University School of Medicine, Boston, MA USA; 3 Department of Molecular and Cell Biology, Goldman School of Dental Medicine, Boston University School of Medicine, 72 East Concord Street, E4, Boston, MA 02118 USA; 4 Department of Pharmacology & Experimental Therapeutics, Boston University School of Medicine, Boston, MA USA; 5 Department of Surgery, Massachusetts General Hospital, Harvard Medical School, Boston, MA USA; 6 Department of Biochemistry, Boston University School of Medicine, Boston, MA USA; 7 Department of Surgery, Brigham and Women’s Hospital, Boston, MA USA; 8 Flow Cytometry Core Facility, Boston University School of Medicine, Boston, MA USA; 9 Department of Microbiology, Boston University School of Medicine, Boston, MA USA

**Keywords:** HNSCC, β-Catenin/CBP transcriptional activity, Aggressive tumor cells, ICG-001, TCGA

## Abstract

**Background:**

Head and neck squamous cell carcinoma (HNSCC) is an aggressive malignancy characterized by tumor heterogeneity, locoregional metastases, and resistance to existing treatments. Although a number of genomic and molecular alterations associated with HNSCC have been identified, they have had limited impact on the clinical management of this disease. To date, few targeted therapies are available for HNSCC, and only a small fraction of patients have benefited from these treatments. A frequent feature of HNSCC is the inappropriate activation of β-catenin that has been implicated in cell survival and in the maintenance and expansion of stem cell-like populations, thought to be the underlying cause of tumor recurrence and resistance to treatment. However, the therapeutic value of targeting β-catenin activity in HNSCC has not been explored.

**Methods:**

We utilized a combination of computational and experimental profiling approaches to examine the effects of blocking the interaction between β-catenin and cAMP-responsive element binding (CREB)-binding protein (CBP) using the small molecule inhibitor ICG-001. We generated and annotated in vitro treatment gene expression signatures of HNSCC cells, derived from human oral squamous cell carcinomas (OSCCs), using microarrays. We validated the anti-tumorigenic activity of ICG-001 in vivo using SCC-derived tumor xenografts in murine models, as well as embryonic zebrafish-based screens of sorted stem cell-like subpopulations. Additionally, ICG-001-inhibition signatures were overlaid with RNA-sequencing data from The Cancer Genome Atlas (TCGA) for human OSCCs to evaluate its association with tumor progression and prognosis.

**Results:**

ICG-001 inhibited HNSCC cell proliferation and tumor growth in cellular and murine models, respectively, while promoting intercellular adhesion and loss of invasive phenotypes. Furthermore, ICG-001 preferentially targeted the ability of subpopulations of stem-like cells to establish metastatic tumors in zebrafish. Significantly, interrogation of the ICG-001 inhibition-associated gene expression signature in the TCGA OSCC human cohort indicated that the targeted β-catenin/CBP transcriptional activity tracked with tumor status, advanced tumor grade, and poor overall patient survival.

**Conclusions:**

Collectively, our results identify β-catenin/CBP interaction as a novel target for anti-HNSCC therapy and provide evidence that derivatives of ICG-001 with enhanced inhibitory activity may serve as an effective strategy to interfere with aggressive features of HNSCC.

**Electronic supplementary material:**

The online version of this article (10.1186/s13073-018-0569-7) contains supplementary material, which is available to authorized users.

## Background

The Wnt/β-catenin signaling pathway plays pivotal roles in development, tissue injury and regeneration, hematopoiesis, and maintenance of somatic stem cell niches in multiple tissue types [1–3]. Under normal conditions of development and homeostasis, the transcriptional activity of β-catenin is subject to negative regulatory controls [4, 5]. In contrast, persistent activation of β-catenin signaling due to somatic inactivating mutations in the upstream regulatory components of the Wnt pathway, or as a result of activating mutations in the β-catenin gene, *CTNNB1*, can contribute to the development of many cancers [1, 6–9].

In head and neck cancer, which presents primarily as head and neck squamous cell carcinoma (HNSCC), mutations in *CTNNB1* are relatively infrequent. Instead, β-catenin activity is induced by the more common mutations in negative regulators of Wnt/β-catenin signaling, specifically in *NOTCH1*, *FAT1*, and *AJUBA* [9, 10], where the inappropriate stabilization of β-catenin has been correlated with de-differentiation and poor prognosis [11]. A large fraction of HNSCC arises in the oral cavity as oral squamous cell carcinoma (OSCC), an aggressive malignancy associated with high morbidity and mortality [12–14]. Although the mechanisms underlying OSCC pathobiology and resistance to therapeutic interventions remain less-understood, mounting evidence suggests that Wnt/β-catenin signaling contributes to advanced OSCC disease and resistance to current therapies [6, 7, 10, 15]. In addition to activating genes with tumor promoting activities, Wnt/β-catenin signaling has been shown to advance aggressive cancer phenotypes through the maintenance of cancer stem cells (CSCs). These CSCs are highly resistant to conventional therapies and are linked to cancer cell expansion, locoregional spread with lymph node metastasis, and tumor recurrence following treatment [16–19]. Recently, CSCs with increased β-catenin transcriptional activity were identified in HNSCC [20], suggesting that targeting β-catenin has the potential to inhibit and eliminate treatment-resistant CSCs, thereby intercepting this malignancy.

The important roles played by Wnt/β-catenin signaling in cancer prompted the development of targeted agents directed at different components of the Wnt/β-catenin pathway. During the past decade, numerous Wnt/β-catenin inhibitors have been tested in preclinical models of different cancers, with some moving on to clinical trials [1, 4, 21]. In particular, several protein and small molecule inhibitors have displayed modest efficacy in vivo [22–24], with those blocking β-catenin activity that impacts its transcriptional targets demonstrating more promise. However, to date, no inhibitors of β-catenin have entered clinical trials for head and neck cancer patients.

In a search for an antagonist of Wnt/β-catenin signaling in OSCC, we have focused on a small molecule inhibitor that preferentially blocks the interaction between β-catenin and cAMP-response element-binding (CREB)-binding protein (CBP) in the nucleus [25]. First identified for its anti-tumorigenic effects in pre-clinical models of colorectal cancer [25], ICG-001 has been shown to have a beneficial role in interfering with multiple cancers and fibrosis [26–30] and to specifically target subpopulations of aggressive HNSCC CSCs [20, 31]. Nonetheless, the activity of ICG-001 has not been investigated in the context of changes in global genomic and cellular programs and their significance to the prognosis and treatment of human HNSCC patients.

To identify an effective strategy to target OSCC cells and to interfere with overall tumor growth and metastasis, we have focused on targeting β-catenin/CBP transcriptional activity using a combination of in vitro and in vivo models coupled with the interrogation of a large high-throughput human OSCC dataset derived from The Cancer Genome Atlas (TCGA). We align molecular and functional approaches with computational interrogation of large data sets for human head and neck cancer cell lines and tumor specimens to comprehensively characterize phenotypic and transcriptional consequences of ICG-001-mediated targeting β-catenin/CBP activity in HNSCC. Our studies provide evidence that inhibition of β-catenin/CBP activity in a panel of OSCC and pharyngeal cell lines by ICG-001 interfered with cell proliferation concomitant with an acquisition of an epithelial-like phenotype. In response to ICG-001, patient OSCC cell line-derived tumor xenografts in nude mice displayed reduced growth and loss of invasive characteristics while acquiring prominent junctional E-cadherin and β-catenin. Further, ICG-001 effectively and preferentially inhibited rapid tumor metastases of aggressive subpopulations of OSCC cells in zebrafish embryos. Importantly, we demonstrate the association between ICG-001 treatment signatures and clinical outcomes in primary human OSCCs. Collectively, our studies align β-catenin/CBP transcriptional activity with OSCC aggressive traits and suggest that targeting β-catenin-CBP interaction represents a potential novel treatment for this malignancy.

## Methods

### Cell lines and EC_50_ measurements

Cell lines were obtained from the following sources: human CAL27, SCC9, and SCC25 cells were purchased from ATCC, and human HSC-3 cells were obtained from XenoTech, Japan. All cell lines were authenticated by short tandem repeat DNA profiling every 6 months either by ATCC or Genetica, with last validation in October, 2016. Cells were grown at 37 °C under 5% CO_2_ to 70% confluence in DMEM (Invitrogen) supplemented with 10% fetal bovine serum, penicillin, and streptomycin, as described [32]. Determination of the half-maximal effective concentration (EC_50_) in response to ICG-001 treatment at different concentrations of the drug was carried out using IncuCyte live-cell imaging per manufacturer’s instructions (Essen BioScience). Briefly, OSCC cells were grown in T75 flasks, collected by trypsin digestion and washed in 5–10 ml DMEM media containing 10% FCS. Cells were seeded in Essen Bioscience 96-well Image Lock plate (Bioscience 96-well ImageLock Microplate no. 4379) at a density of 2000–3000 cells/well and allowed to attach for 12–24 h. Next, cells were treated with different concentrations of ICG-001 (Selleckchem):0.1, 0.3, 1, 3, 10, 20, and 30 μM and examined at 6-h time intervals. After 72 h, data were transferred to the Excel format and analyzed using the GraphPad Prism program.

### Nude mice and orthotopic xenograft experiments

HSC-3 cells were lentivirally transduced with DsRed and suspended in serum-free Dulbecco’s Modified Eagle Medium (DMEM). For orthotopic tongue xenografts, 0.5 × 10^6^ cells in 40 μl of DMEM were injected into each tongue of 2-month-old nude mice (Taconic Farms, Hudson, NY) that were anesthetized with isoflurane. Anesthesia was administered in an induction chamber with 2.5% isoflurane in 100% oxygen at a flow rate of 1 L/min and then maintained with a 1.5% mixture at 0.5 L/min. Two days following injection, mice (*n* = 16) were equally divided into two groups: vehicle (DMSO control) and ICG-001-treated. For tumor inhibition studies, ICG-001 was diluted in 0.1% DMSO and administered via oral gavage at a concentration of 80 mg/kg into treatment-group mice via the tongue daily. Digital caliper measurements were performed at 2–3 day intervals to monitor the volumes of all tumors. Mice were imaged for DsRed protein expression on day 17 using an IVIS 200 system (Xenogen, Alameda, CA, USA). The fluorescence signals were optimized for the DsRed protein at excitation 570 and emission 620. Fluorescence region of interest (ROI) data were calibrated and normalized fluorescence efficiency (p/s/cm^2^/sr)/(μW/cm^2^) determined as per the instructions (Perkin Elmer, USA). The data are reported as normalized fluorescence intensity (FU) from a defined region of interest for oral tongue tumors or systemic metastases compared to control vehicle-injected mice. Mice were sacrificed at day 20, and tumors were harvested and processed for further analyses.

### Immunocompetent SCC mouse model

HPV-16 E6 and E7-expressing TC-1 tumor cells were used which were generated as previously described [33]. In brief, the HPV-16 E6, E7, and *ras* oncogene were used to transform primary C57BL/6 mice lung epithelial cells to generate the TC-1 tumor cell line. TC-1 has been reliably used as a preclinical model to study HPV16 E7-specific CD8^+^ T cell responses for the evaluation of novel immunotherapeutic agents to support phase I clinical trials in human subjects [34]. The cells were maintained in RPMI medium supplemented with 2 mM glutamine, 1 mM sodium pyruvate, 100 U/ml penicillin, 100 lg/ml streptomycin, and 10% fetal bovine serum. C57BL/6 mice were inoculated subcutaneously in the right flank with 1 × 10^5^ TC-1 cells per mouse. When the tumor volume reached 50 mm^3^ (7 days post-inoculation), mice were treated daily with either 50 μl of ICG-001 (0.04 mg/μl; 80 mg/kg BW) or a mixture of 10% DMSO + 90% vegetable oil (vehicle control group) administered orally using a 16-gauge oral feeding tube daily for 7 days. Mice were sacrificed, and tumors were dissected and processed as described below.

### Mouse tumor sample processing

Tumors were harvested at sacrifice at day 20, weighed and either snap-frozen, and processed for histology, immunohistochemistry, immunoblot, and flow cytometry analyses. Tumors were paraffin-embedded and 5 μm sections were placed on Fisherbrand Superfrost Plus Microscope Slides (Fisher Scientific), deparaffinized, treated with Retrievit-6 Target Retrieval Solution (BioGenex), and processed for immunofluorescence and histological analyses (see below). Frozen tumor tissues were used for immunoblot analyses. In addition, portions of mouse tumors were minced and digested with 0.1% collagenase I (Worthington) at 37 °C for 1 h. Cells were passed through 70 μm mesh and washed twice, and the resulting single-cell suspension was used for flow cytometric analysis and sorting.

### Immunofluorescence analysis

Morphologies of OSCC cell lines comprising CAL27, HSC-3, SCC9, and SCC25 cells treated with ICG-001 or DMSO vehicle control were examined using a Nikon Eclipse TE300 microscope. For indirect immunofluorescence analyses, cells were grown to 70% confluence on Nunc Lab-Tek II Chamber Slide (Thermo Fisher Scientific), fixed in 3.7% paraformaldehyde, permeabilized with 0.1% Triton X-100, blocked with 10% goat serum, and incubated with primary antibodies to β-catenin (Abcam, rabbit polyclonal AB followed by secondary antibody, goat anti-rabbit conjugated with Alexa Fluor 488 (Jackson ImmunoResearch). Cells were counterstained for nuclei with 4′6-diamidino-2-phenylindole, dihydrochloride (DAPI) (Molecular Probes), mounted in ProLong Gold Antifade, (Molecular Probes), and images were analyzed with a Zeiss LSM 710-Live Duo Scan confocal microscope. The observed changes in cellular phenotypes in response to the ICG-001 treatment were quantified using two independent criteria: cell size (area occupied by cell, *n* = 15) and junctional organization of β-catenin at the membrane (width of membrane localization, *n* = 10). All images were quantified using the ImageJ software. Mouse tumor tissue sections were blocked with 10% goat serum, mouse IgG blocking reagent (Vector) and incubated with antibodies against β-catenin, E-cadherin (Abcam, rabbit), and vimentin (Sigma, mouse) followed by secondary antibodies, goat anti-rabbit and goat anti-mouse conjugated with Alexa Fluor 488. Negative controls lacked primary antibodies. The slides were mounted in ProLong Gold Antifade, and optical sections (0.5 μm intervals) were analyzed by confocal microscopy using a Zeiss LSM710-Live Due Scan confocal microscope. To compare fluorescence intensities between samples, settings were fixed to the most highly stained sample with all other images acquired at those settings. Images were processed with ZEN 2 and ImageJ imaging software.

### Immunoblot analysis

Total tissue lysate (TTL) from harvested mouse orthotopic tongue tumors was prepared by grinding tumors to a fine powder in liquid nitrogen and extracting total tissue proteins with Triton/β-octylglucoside buffer. Protein concentrations for TTL were determined using BCA assay (Pierce). For immunoblot analyses, TTL (20–30 μg of total protein) were fractionated on 7.5% SDS-PAGE, transferred onto polyvinylidene difluoride membranes, blocked with 5% nonfat dry milk, and incubated with primary antibodies to either E-cadherin or β-catenin (Abcam, rabbit) and GAPDH (Novus Biologicals). Protein-specific detection was carried out with horseradish peroxidase-labeled secondary antibodies conjugated to horseradish peroxidase (Bio-Rad) and Enhanced Chemiluminescence Plus (Amersham Biosciences). Signal intensities were normalized to GAPDH (Novus Biologicals).

### Histology

Mouse tumor tissues were fixed overnight in 4% paraformaldehyde in PBS and dehydrated. Samples were embedded in paraffin and sectioned (5 μm thickness) and stained with hematoxylin and eosin (H&E) at the Boston University Medical Campus Pathology Core Facility. Digital images of stained slides were acquired with a TE 300 microscope (Nikon). Quantification of the effects of ICG-001 on tumor spread in harvested tongue tumors was carried out by measuring tumor area (region occupied by cancer cells) relative to the total section on the slide.

### Zebrafish transplantation and ICG-001 treatment

Zebrafish husbandry was performed as described [35]. *Casper x Fli-GFP* fish breeders were crossed, and embryos overexpressing GFP in their vasculature were obtained. For micro-injections of zebrafish embryos, OSCC cells were either stained with a CellTracker™ dye (Molecular Probes) or lentivirally transduced with red fluorescent protein (RFP), trypsinized, and resuspended at a concentration of 50 × 10^6^ cells/ml in DMEM containing 10% FBS. One day post-fertilization (dpf) embryos were enzymatically dechorionated using Pronase (Roche Diagnostics) and allowed to recover overnight at 28 °C, in the dark and in sterilized egg water. The two-dpf zebrafish larvae were anesthetized with Tricaine and immobilized before injections. The borosilicate glass capillaries (World Precision Instruments) 1.0 mm O.D. ×  0,78 mm (I.D. Harvard Apparatus) used for micro-injection were pulled using 500 V (pull = 100, velocity = 250) in a capillary machine (Sutter Instrument)*.* The OSCC cell transplantations were performed by injection of ~ 1 nL directly into the perivitelline space [36] of the embryo using a needle holder and a micro-injection station (World Precision Instruments)*.* The transplanted zebrafish embryos were treated with ICG-001 or vehicle and incubated in the dark at 34.6 °C. Zebrafish embryos were individually mounted in a low melting 1.5% agarose gel (Fisher BioReagents) for a side view and imaged using an Olympus MVX10 fluorescence stereomacroscope or a Leica SP5 laser scanning confocal microscope at Boston University cellular imaging core. For comparison of metastases in CAL27 and HSC-3 cell-injected fish, metastases were measured by scoring numbers of CellTracker™-labeled fish outside the injection site. To quantify the extent of metastases in zebrafish injected with RFP-labeled HSC-3 cells, individual fish were scored for numbers of cells.

### Flow cytometry and fluorescence-activated cell sorting (FACS)

For characterization of cells from immunocompetent SCC mouse model by multicolor flow cytometry staining, cells were first incubated with Zombie Aqua Live-dead dye (Biolegend) in PBS for 20 min, washed, pre-blocked with mouse antiCD16/32 FcBlock (Biolegend), and stained with the cocktail of conjugated fluorescent anti-mouse antibodies: CD29 eFluor 450, CD166 PE and CD133 PerCP-e710 (eBioscience), CD44 BV786, EpCAM FITC, E-Cadherin PE-Cy7, CD24 Alexa Fluor 647, and CD45 APC-750 (BioLegend) for 30 min. Cells were washed and immediately run on the BD FACSARIA II flow cytometry sorter for sorting and analysis. At least 200,000 events were recorded per sample. Data were analyzed in FACSDiva v6.2.0 (BD) and FlowJo v10.2 (FlowJo).

For zebrafish studies, cells were prepared for flow cytometry as a single-cell suspension and stained with Zombie Aqua Live-Dead dye (Biolegend) in DPBS for 20 min in the dark according to manufacturer’s protocol, then washed and pelleted by centrifugation. The cell pellet was resuspended in DPBS/BSA/EDTA buffer containing Brilliant Buffer (BD Biosciences) and fluorochrome-conjugated antibodies to human CD29 (Alexa Fluor 700, Biolegend), human CD24 (BUV395, BD Biosciences), human EpCAM (BV650, BD Biosciences), and human CD44 (BV421, Biolegend), incubated for 30 min in the dark, then washed and pelleted twice. The resulting cell pellet was resuspended in DPBS/BSA/EDTA buffer and immediately analyzed on a BD FACSAria II SORP sorter located in the Flow Cytometry Core Facility at Boston University Medical Center (BUMC) using the BD FACSDiva 6.2 software. For analysis, at least 100,000 events per sample were recorded and data were subsequently analyzed with FlowJo 10.2 software (FlowJo, Inc). For quality controls, purity of the sorts was confirmed to be > 95%.

### Microarray gene expression analysis

Three separate wells (triplicate) from 6-well plates were seeded by HSC-3 and CAL27 cells (around 5 × 10^4^/well). After 12–24 h (i.e., approximately 50% confluency), the cells were treated with either 0.1% DMSO or 10 μM ICG-001. After 48–64 h, the cells were trypsinized and harvested for RNA purification using miRNeasy Micro Kit (QIAGEN no. 217084). Gene expression profiling was performed in triplicates per treatment group (*n* = 3) using Affymetrix Human Gene 2.0ST arrays. Raw expression values were normalized together using the Robust Multiarray Average (RMA) with the affy R package. A BrainArray Chip Definition File (CDF) was used to map the probes on the array to unique Entrez Gene identifiers (http://brainarray.mbni.med.umich.edu/Brainarray/Database/CustomCDF). Differential gene expression analysis with respect to ICG-001 treatment was performed for each cell line using the Limma R package v3.14.4 [37]. Filtered false discovery rate (FDR) *q* values were recomputed based on the Benjamini-Hochberg method [38] after removing genes that were not expressed above the array-wise median value of at least one array. Only genes with absolute linear fold-change ≥ 1.5 and filtered FDR *q* ≤ 0.01 were considered and used for further analysis as the ICG-001 treatment signature.

### CCLE and TCGA data processing and analysis

Microarray gene expression data from the Cancer Cell Line Encyclopedia (CCLE) was processed as previously described [39]. Only data for cell lines annotated as originating in the upper aerodigestive tract (UAT; *n* = 32) was used. RNA-sequencing (RNASeq) expression and matched clinical data pertaining to The Cancer Genome Atlas (TCGA) OSCC samples (*n* = 352) was obtained and processed as previously described [40]. For gene set projection analyses, TCGA OSCC RNASeq and CCLE UAT microarray data were separately projected onto the “core” set of genes downregulated by ICG-001 in both HSC-3 and CAL27 cells (*n* = 104) using ASSIGN [41], yielding sample stratification based on a score representing the coordinated expression of ICG-001-downregulated genes (referred to as the ICG-001-*inhibition* score). For TCGA OSCCs, only samples with known tumor grade information were retained when comparing scores with respect to tumor grade. Survival analysis was performed using the survival package in R (https://cran.r-project.org/). Patient survival information pertaining to the February 4th 2015 Firehose release was used to compute overall survival for TCGA OSCC patients. Patients were divided into two groups (ICG-001-high and -low) based on the median ICG-001 inhibition scores. AE and metastatic tumor samples were filtered out for this analysis (i.e., only the primary tumor-associated inhibition scores were used to bin samples into ICG-001 high, and low groups).

### Statistical analyses

Statistics for all pairwise comparisons of quantitative measurements, including relative abundances from TTL obtained using immunoblot assays, tumor volume, relative tumor area within harvested tumor sections, and relative radiant efficiency measurements in orthotopic mice, as well as metastases quantification in injected zebrafish, were obtained using an unpaired two-tailed *t* test between comparison groups (ICG-001 versus DMSO vehicle, unsorted versus sorted cells etc.). Mouse tumor volume measurements were compared between groups per time point. All bar plots are represented as means ± S.D. ICG-001 ASSIGN scores for OSCC versus AE TCGA samples were compared using an unpaired two-tailed *t* test. Analysis of scores with respect to tumor grade was done using a pairwise one-sided *t* test between adjacent grade groups (g2 versus g1, g3 versus g2, and g4 versus g3), with their corresponding *p* values combined as previously described [42]. A log-rank test was used to compare survival rates between the ICG-001-high and ICG-001-low patient groups.

## Results

### Inhibition of β-catenin/CBP-activity interferes with OSCC cell growth and promotes membrane localization of β-catenin

**Fig. 1:**
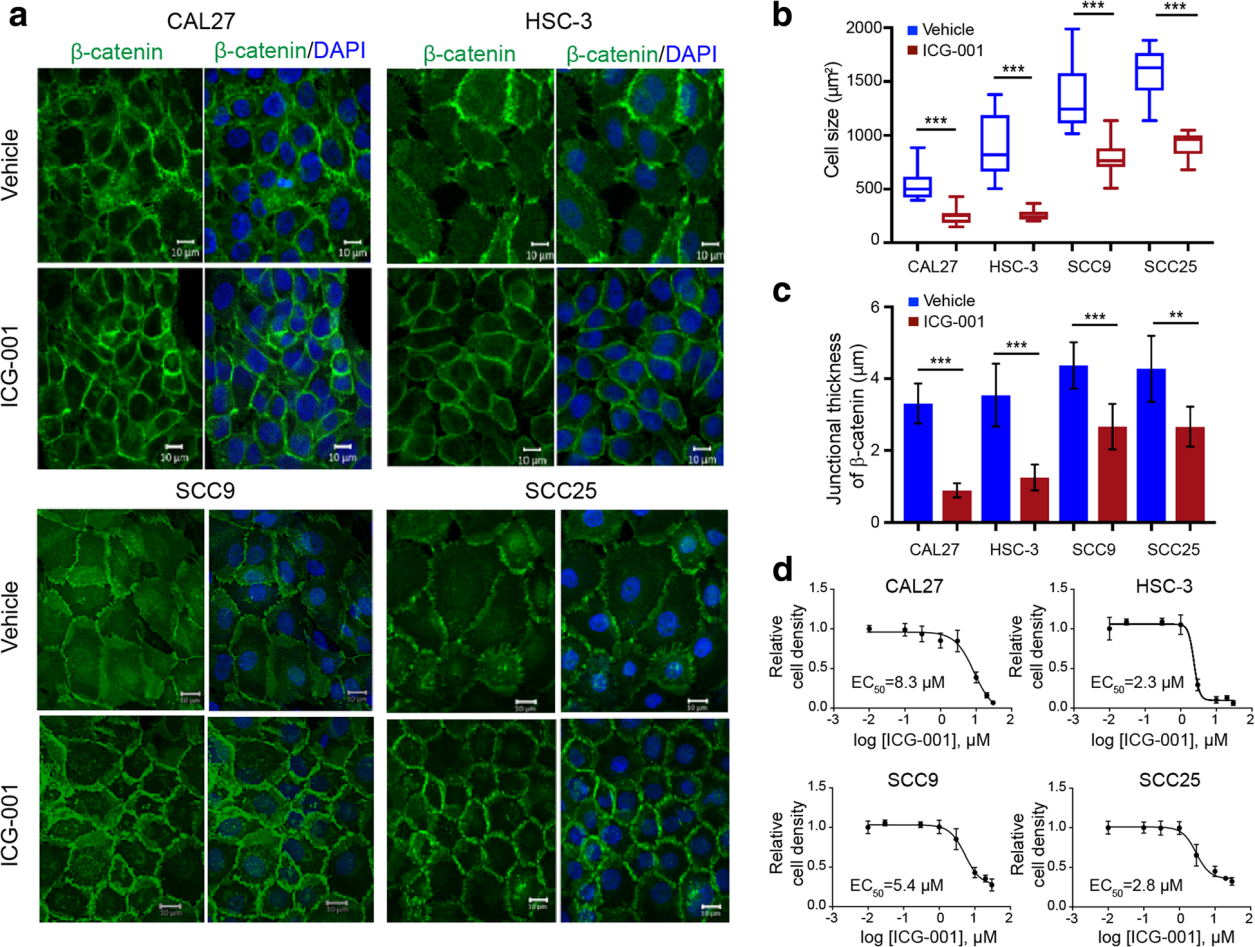
ICG-001 inhibits growth of OSCC cell lines and promotes an epithelial phenotype. **a** Immunofluorescence imaging of β-catenin localization in OSCC cell lines in response to ICG-001 treatment showing more focused membrane localization of β-catenin, increased intercellular adhesion with a more pronounced epithelial phenotype. Size bars, 10 μm. **b** ICG-001 treatment promotes cell compaction as measured by the average cell size (*n* = 15) captured in 1a. **c** Increased junctional organization of β-catenin in immunofluorescence images shown in 1a was determined based on the thickness of β-catenin at the membrane (*n* = 10). **d** Determination of half maximal effective concentration (EC_50_) for ICG-001 effects in OSCC cell lines. Shown are graded dose response curves for ICG-001 in CAL27, HSC-3, SCC9, and SCC25 cell lines for 72 h of treatment. Immunofluorescence images were quantified using ImageJ and compared using PRISM GraphPad. **P < 0.001; ***P < 0.0001 unpaired *t* test

The small molecule inhibitor, ICG-001, has been shown to inhibit the interaction between β-catenin and CBP in the nucleus and to affect transcription of a subset of β-catenin target genes [25, 43]. We first verified the inhibitory effect of ICG-001 on transcriptional activity of β-catenin using the TOPflash-driven Renilla luciferase reporter assay (Additional file 1: Figure S1). Given that intrinsic activity of β-catenin has been associated with treatment-resistant features of carcinomas, we examined whether ICG-001 would impact phenotypes of OSCC cells. Thus, we examined β-catenin localization in response to ICG-001 in a panel of cell lines including CAL27, HSC-3, SCC9, and SCC25 cells using immunofluorescence staining and high-resolution confocal microscopy. Remarkably, while vehicle-treated cells displayed a broad distribution of β-catenin at the membrane, ICG-001 promoted a more focused localization of β-catenin in all four cell lines evaluated (Fig. 1a). This was accompanied by a more compact epithelial cell phenotype, as indicated by reduced average cell size (Fig. 1b). Additionally, examination of junctional β-catenin by immunofluorescence imaging in Fig. 1a revealed tighter organization as indicated by its diminished thickness at the membrane, a feature that accompanies more mature E-cadherin junctions (Fig. 1c). To better visualize the effects of ICG-001 on β-catenin localization and OSCC cell morphology, we imaged CAL27 cells at a higher power and found that in vehicle-treated cells, β-catenin was localized at widespread membrane projections that formed loose cell-cell contacts. These cells displayed a mesenchymal morphology with prominent stress fibers and little co-localization between β-catenin and F-actin at the membrane (Additional file 1: Figure S2a, DMSO). In contrast, in ICG-001-treated cells, β-catenin was enriched at membrane domains with reduced projections, where it co-localized with F-actin (Additional file 1: Figure S2a, ICG-001). The differences in cell size and β-catenin junctional organization in response to ICG-001 treatment in CAL27 and HSC-3 cells did not involve major changes in E-cadherin and β-catenin transcript or protein levels (Additional file 1: Figure S2b), suggesting altered distribution of these components rather than expression. Instead, we found that the CBP abundance was substantially reduced following the ICG-001 treatment (Additional file 1: Figure S2b), a result supported by the fractionation of β-catenin and CBP from HSC-3 cells into cytoplasmic and nuclear fractions (Additional file 1: Figure S3). Interestingly, with this approach, we detected majority of β-catenin in the cytosolic compartment with only a small fraction in the nucleus. Collectively, these results strongly suggest that treatment of OSCC cells with ICG-001 promoted junctional localization of β-catenin and enhanced their epithelial-like morphology.

We next determined the in vitro sensitivity of the four OSCC cell lines to ICG-001 using incuCyte live imaging. Dose-dependent inhibition of cell growth was observed for all cell lines examined following 72 h of treatment with ICG-001, with EC_50_ values of 2.3, 2.8, 5.4, and 8.3 μM for HSC-3, SCC25, SCC9, and CAL27 cells, respectively (Fig. 1d). Notably, the most aggressive metastatic cell line exhibited the greatest sensitivity to ICG-001, especially when compared to the non-metastatic CAL27 cells (Fig. 1d).

### ICG-001 impacts expression of genes involved in Wnt signaling, cell proliferation, survival, and intercellular adhesion

**Fig. 2:**
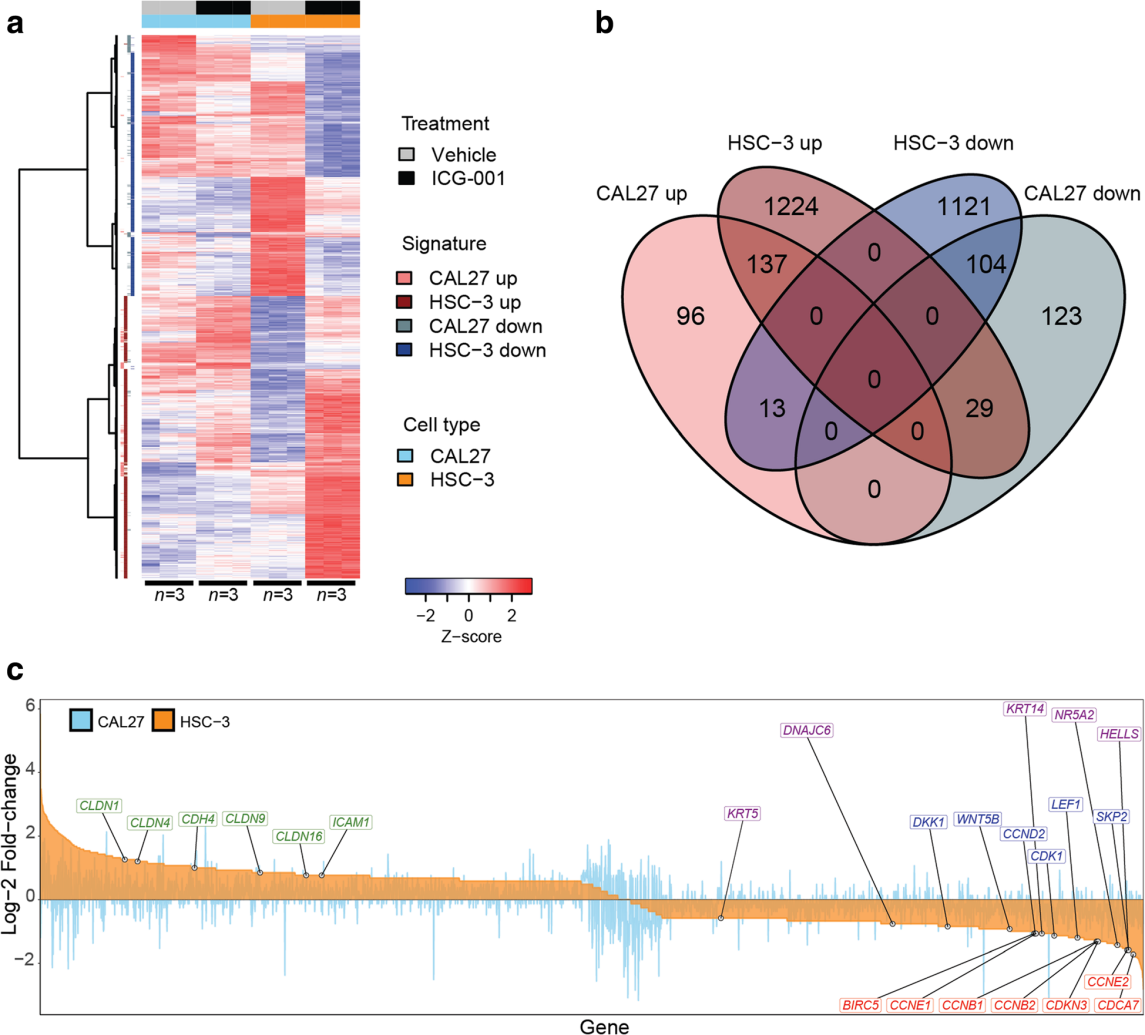
Genome-wide transcriptional profiling of non-metastatic and metastatic OSCC cells treated with ICG-001. **a** Heatmap of microarray gene expression treatment signatures derived from metastatic HSC-3 and non-metastatic CAL27 cells. RNA was extracted and profiled using microarrays from each cell line and gene expression profiles were compared by differential expression testing (ICG-001 treatment versus DMSO vehicle; *n* = 3 per treatment group, per cell line). Signatures were derived for each cell line by retaining genes with filtered-FDR *q* ≤ 0.01 and absolute linear fold-change ≥ 1.5. Row *z* scores of log_2_ expression values per gene are shown. **b** Metastatic HSC-3 cells display higher sensitivity to transcriptional dysregulation by ICG-001 treatment than less aggressive CAL27 cells. Venn diagram summarizing the size and overlap of treatment signatures in HSC-3 and CAL27 cells is shown. **c** ICG-001 impacts the expression of genes with roles in Wnt/β-catenin signaling (blue), cell proliferation and survival (red), intercellular adhesion (green), and stem cell-like behavior (purple). Waterfall plot displaying log_2_ fold-changes of gene expression for all genes included in either HSC-3 or CAL27 treatment signatures (*n* = 2847 genes) is shown

**Fig. 3:**
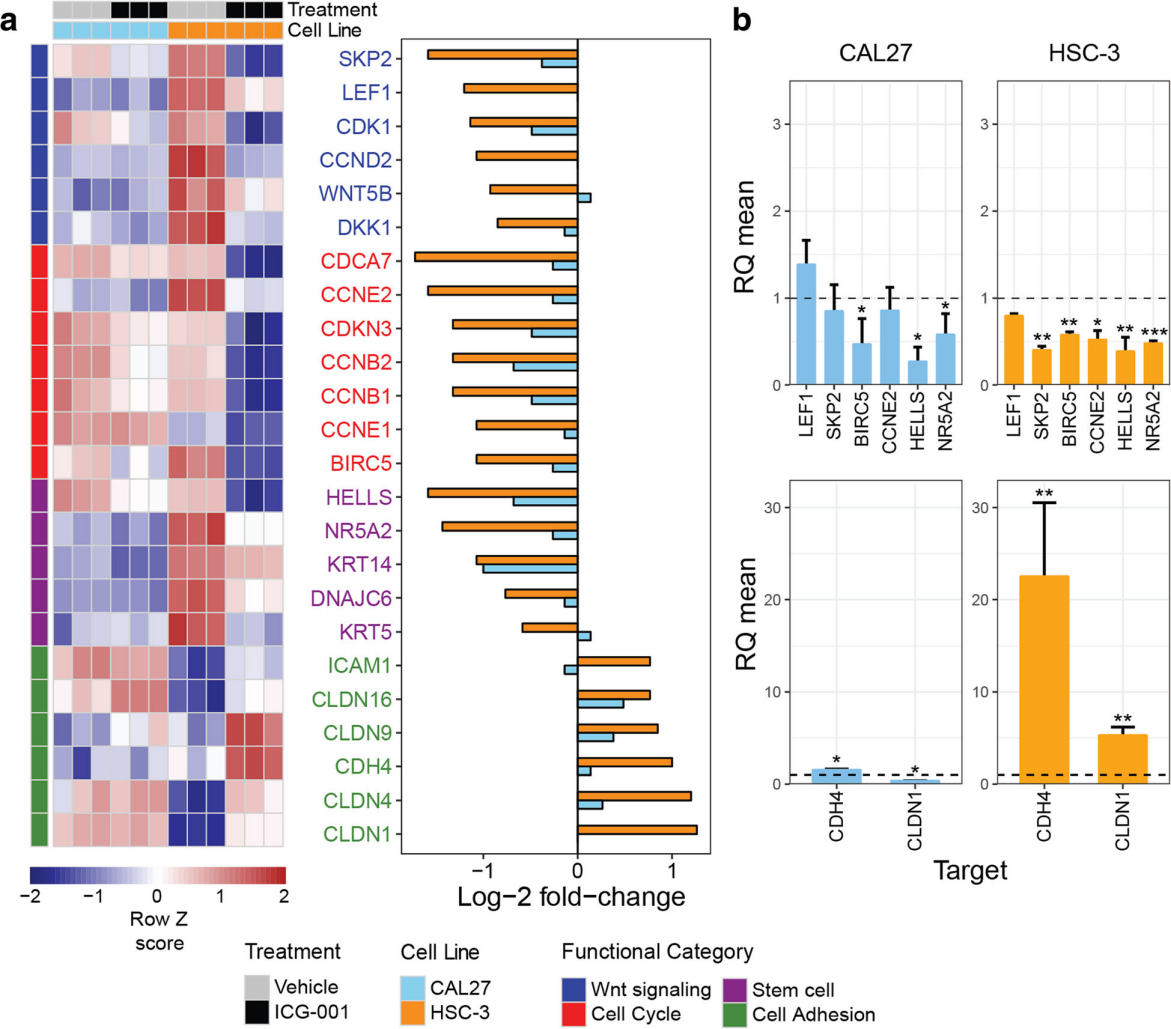
Differential expression profiles and validation of key genes affected by ICG-001 in OSCC cells. **a** Heatmap of microarray gene expression levels pertaining to genes highlighted in Fig. 2c, along with their log fold-change of expression upon ICG-001 treatment in HSC-3 and CAL27 cells. **b** qRT-PCR validation of microarray profiles for select target genes confirming changes in gene expression upon treatment with ICG-001. *P < 0.05; **P < 0.01; ***P < 0.001 unpaired *t* test

For the subsequent studies, we selected CAL27 and HSC-3 cells to represent non-metastatic and metastatic OSCC phenotypes, respectively. To determine the effects of disrupting β-catenin/CBP-mediated activity with ICG-001 in OSCC cell lines, we performed gene expression profiling of RNA isolated from CAL27 and HSC-3 cells treated with either vehicle (DMSO) control or ICG-001 using microarrays. Differential gene expression testing between ICG-001 treatment and control groups yielded cell type-specific ICG-001 treatment signatures (Fig. 2a and Additional file 2). Consistent with EC_50_ values in vitro, which demonstrated greater ICG-001 treatment sensitivity of HSC-3 cells as compared to CAL27, HSC-3 cells showed a greater transcriptional response to treatment (1390 upregulated and 1238 downregulated genes) than CAL27 cells (246 upregulated and 256 downregulated genes) (Fig. 2b). Importantly, genes known to be targets of Wnt/β-catenin signaling (*DKK1*, *WNT5B*, *CCND2*, *CDK1*, *LEF1*, and *SKP2*) and to play a role in cell survival and proliferation (*BIRC5*, *CCNE1*, *CCNE2*, *CCNB1*, *CCNB2*, *CDKN3*, and *CDCA7*) were significantly downregulated, while genes with key roles in intercellular adhesion and epithelial differenetiation (*CLDN1*, *CLDN4*, *CLDN9*, *CLDN16*, *CDH4*, and *ICAM1*) were upregulated, with greater effect in HSC-3 cells than in CAL27 cells (Fig. 2c and Fig. 3a). Of significance, cell markers that were previously identified to be associated with a more stem cell-like state in HNSCC (*DNAJC6*, *NR5A2*, *HELLS*, *KRT5*, and *KRT14*), and characterized by elevated β-catenin activity [20, 44], were also found to be significantly downregulated by ICG-001 in HSC-3 cells (Fig. 2c and Fig. 3a). Treatment effects of ICG-001 on selected genes within these functional groups in CAL27 and HSC-3 cells were confirmed using quantitative real-time PCR (Fig. 3b). This includes the significant upregulation of cell-cell adhesion genes in HSC-3 cells, *CDH4* and *CLDN1*, further supporting the observed phenotypic effects of ICG-001 (Fig. 1a–c) and convergence between Wnt/β-catenin signaling and intercellular adhesion [45]. Immunoblot analyses of protein products of genes impacted by ICG-001 in CAL27 and HSC-3 revealed reduction in the steady-state levels of survivin, the *BIRC5* gene protein product, and products of genes associated with stem cell-like-phenotypes, *HELLS* and *KRT14* (Additional file 1: Figures S4 and S5). Also, expression of a differentiation marker, claudin 1, was increased substantially in HSC-3 cells, while it was not altered in non-malignant CAL27 cells consistent with no significant changes detected in *CLDN1* transcript levels in CAL27 cells (Additional file 1: Figures S4 and S5).

To examine if the inhibition of β-catenin/CBP activity by ICG-001 has a broader relevance, we aligned the activity of genes repressed by ICG-001 in CAL27 and HSC-3 cells relative to additional cancer cell lines. Specifically, we interrogated gene expression data pertaining to a “core signature” comprising genes significantly downregulated by ICG-001 in both HSC-3 and CAL27 cells (*n* = 104 genes; Fig. 2b) in a panel of UAT cell lines from the CCLE (see “Methods”). Using the ASSIGN algorithm [41], we projected gene expression data for these cells in the space of the core gene signature, scoring each sample based on the coordinated expression of the gene set (referred to as the “ICG-001 inhibition score”; Additional file 1: Figure S6). Of the panel of OSCC cell lines assessed for sensitivity to ICG-001 treatment (Fig. 1d), HSC-3 cells displayed the highest ICG-001 inhibition score, followed by SCC25 cells, similar the trend observed based on EC_50_ values. Further, as per EC_50_ values, CAL27 and SCC9 cells were ranked lower (Fig. 1d; Additional file 1: Figure S6). In addition, we observed that hypopharygeal SCC FaDu cells clustered next to HSC-3 cells (rank = 1/32), suggesting that, although these cells are derived from a different anatomic site, they may also exhibit sensitivity to ICG-001 treatment. While FaDu cells did show sensitivity to treatment (EC_50_ = 3.5 μM; Additional file 1: Figure S7), it was lower compared to the EC_50_ values for HSC-3 and SCC25 cells, most likely reflecting differences arising from cell-specific and anatomical site-specific treatment effects. Importantly, treatment with ICG-001 of FaDu cells led to downregulation of CBP steady-state levels, consistent with CAL27 and HSC-3 cells (Additional file 1: Figure S7). In addition, levels of survivin were reduced in response to ICG-001 in FaDu cells. However, we did not detect changes in proteins encoded by *HELLS* and *CLDN1*, while the protein product of *KRT14* was not detectable.

To assess whether transcriptional effects of ICG-001 treatment correlated with that induced by β-catenin knockdown in OSCC in vitro, we first generated a gene list ranked by differential expression of β-catenin siRNA knockdown (KD) versus control in HSC-3 cells (Additional file 3). We then queried the derived ICG-001 treatment signatures against this reference list using Gene Set Enrichment Analysis (GSEA; see Additional file 1 for methods). We observed a significant skewedness of ICG-001 down and upregulated genes towards negative and positive fold-change of gene expression associated with β-catenin KD, respectively (FDR *q* < 0.001; Additional file 1: Figure S8a), suggesting that the ICG-001 treatment targeted specific β-catenin-mediated transcriptional activity in aggressive OSCC cells.

### ICG-001 inhibits OSCC tumor growth and aggressive phenotypes in vivo

**Fig. 4:**
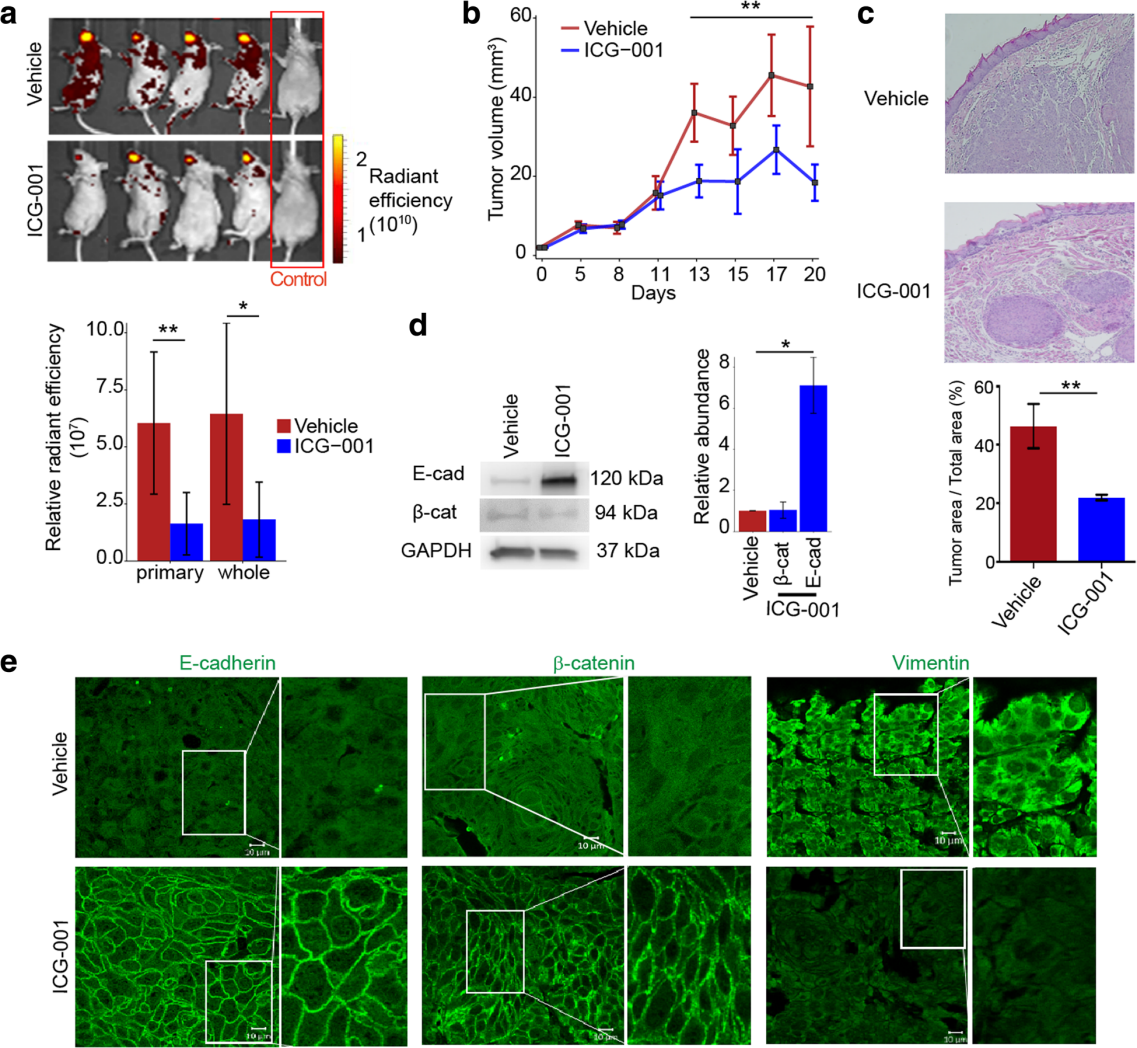
ICG-001 inhibits OSCC growth and metastasis in vivo. **a** HSC-3 cells (0.5 × 10^6^ cells/40 μl DMEM) were lentivirally transduced with DsRed and injected into tongues of 2-month-old nude mice. Tumor formation and metastasis were visualized using IVIS fluorescent imaging at day 17; representative images for mice treated with ICG-001 (*n* = 8; bottom) or DMSO vehicle (*n* = 8; top) and for control mice (*n* = 2) are shown. Total radiant efficiency of cells in the primary tongue tumor and of cells that metastasized to other organs was significantly reduced upon ICG-001 treatment for both the primary tongue tumor and whole body metastases (shown as the mean ± S.D.). **b** Caliper measurements confirmed significantly reduced primary tumor volume in ICG-001- compared to DMSO-treated mice starting at day 13 with continued trend until sacrifice at day 20. **c** Harvested tumors were analyzed by H&E staining for differences in morphologies. ICG-001-treated tumors exhibited a capsular phenotype, in contrast to untreated tumors that showed streaks of invasive cells growing into the tongue stroma. Fraction of the total tissue taken up by invasive epithelial cells was quantified for vehicle- and ICG-001-treated tumors (*n* = 3 per treatment group). **d** Total tissue lysates were prepared from mouse tumors (*n* = 4 per treatment group) and analyzed by immunoblot for E-cadherin and β-catenin protein levels after normalization to GAPDH. Immunoblot and bar graphs displaying relative protein levels are shown. **e** Treatment with ICG-001 alters localization of key epithelial and mesenchymal markers. Immunofluorescence imaging of E-cadherin, β-catenin, and vimentin in tumors treated with either DMSO vehicle (top) or ICG-001 (bottom). ICG-001 enhanced the recruitment of E-cadherin and β-catenin to cell-cell contacts, while reducing the expression of vimentin. *P < 0.05; **P ≤ 0.005; unpaired *t* test

Our previous studies have shown that while CAL27 cells form orthotopic tongue tumors in nude mice, these tumors do not metastasize to distant sites. In contrast, HSC-3 cell-driven tumors undergo significant metastases in nude mice [46]. To determine whether ICG-001 treatment impacted growth and metastasis of HSC-3 xenografts, we treated mice (*n* = 16) with either ICG-001 (*n* = 8) or DMSO vehicle (*n* = 8) by oral gavage beginning on day 3 following cell implantation into the tongues of nude mice, with treatment repeated every day. For these studies, cells were transduced with red fluorescent protein (DsRed) to track tumor growth and metastasis prior to orthotopic inoculation. Primary tumor growth was monitored every 2–3 days using caliper measurements. In addition, in vivo imaging system (IVIS) was used to follow localization of DsRed-expressing cells. Comparison of relative radiance efficiencies at day 17 post-inoculation revealed significantly reduced primary tumor growth (*P* = 4.65e−3, two-tailed *t* test) and whole body metastases (*P* = 0.013, two-tailed *t* test) associated radiance intensities in the treated versus control groups (Fig. 4a). Further, starting at 13 days post-inoculation, we observed a significant reduction in tumor volume (*P* < 0.005, two-tailed *t* test) following treatment with ICG-001, relative to vehicle treated-control mice (Fig. 4b). Histological hematoxylin and eosin (H&E) analysis of vehicle-treated tumors revealed streaks of invasive cells growing out from primary tumors into the tongue stroma, while ICG-001-treated tumors displayed a capsular phenotype (Fig. 4c). Quantification of the tumor cross-sectional area taken up by squamous carcinoma cells was significantly reduced relative to the control condition (*P* = 0.005, two-tailed *t* test; Fig. 4c). Interestingly, examination of E-cadherin protein levels by immunoblot revealed that ICG-001-treated tumors exhibited increased E-cadherin abundance compared to vehicle control-treated tumors (*P* = 0.016, two-tailed *t* test), while levels of β-catenin were not significantly altered (*P* = 0.67, two-tailed *t* test; Fig. 4d). Immunofluorescence analyses of these tumors showed that the ICG-001-treated tumors phenocopied ICG-001-treated OSCC cell lines in vitro and displayed robust E-cadherin and β-catenin junctional localization with an almost complete loss of a mesenchymal protein vimentin, further indicating that ICG-001 promoted an epithelial phenotype (Fig. 4e).

**Fig. 5:**
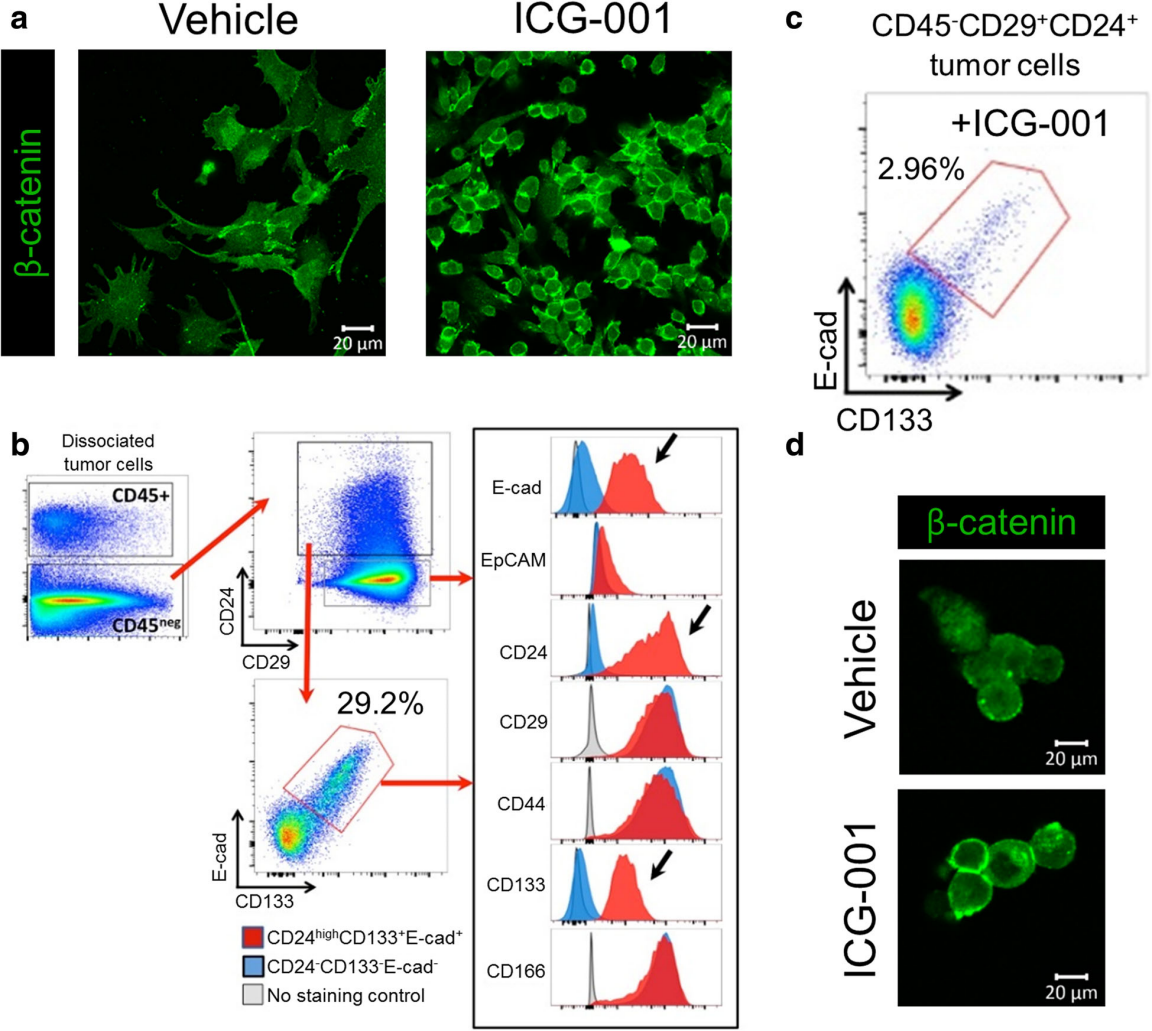
ICG-001 inhibits aggressive phenotypes and promotes membrane localization of β-catenin in an immunocompetent mouse model of SCC. **a** Immunofluorescence imaging of β-catenin in HPV-16 E6 and E7-expressing TC-1 tumor cells. ICG-001 induces a cuboidal phenotype in cells dissociated from TC-1 tumors and promotes expression of junctional β-catenin. Representative images are shown for ICG-001 treatment versus DMSO (vehicle) control conditions. **b** Flow cytometry phenotyping of TC-1 tumor cells. Live, single cells were further gated based on expression of CD45, CD24, CD29, CD133, and E-cadherin (E-cad) as shown (red arrows), and expression profiles of stem cell-like CD24^high^CD133^+^E-cad^+^ (red histograms, black arrows) were compared to CD24^−^CD133^−^E-cad^−^ (blue histograms). **c** Stem cell-like CD24^high^CD133^+^E-cad^+^ population in ICG-001-treated group is greatly diminished. Gating was performed as shown in panel **b**. **d** Localization of β-catenin in FACS-sorted CD24^high^CD133^+^E-cad^+^ cells. Treatment of CD24^high^CD133^+^E-cad^+^ cells with ICG-001 resulted in increased membrane distribution of β-catenin compared to vehicle-treated cells

Given that our in vivo model was based on an immunocompromised mouse, it was possible that the observed tumor-inhibitory effects of ICG-001 were enhanced by the absence of the immune system. We therefore further tested whether ICG-001 inhibited aggressive phenotypes and promoted membrane localization of β-catenin in an immunocompetent mouse model of squamous cell carcinoma (SCC) [47]. Specifically, we assessed the effects of ICG-001 treatment on lung squamous cell carcinoma TC-1-derived tumor xenografts from syngeneic C57BL/6 mice. Strikingly, we found that treatment with ICG-001 completely abrogated aggressive mesenchymal-like phenotypes of dissociated tumor cells coincident with the acquisition of a cuboidal epithelial-like morphology (Fig. 5a). We next searched for subpopulations of cells with immature cell surface markers from TC-1-derived tumors by flow cytometry using a panel of primitive epithelial cell surface markers that included CD44, CD29, CD24, CD166, and CD133. Phenotyping these tumor cells identified a subpopulation of CD29^+^ and E-cadherin-expressing (CD29^+^E-cad^+^) cells enriched for cell surface antigenic markers of primitive cell phenotypes, namely CD24 and CD133 (CD24^high^CD133^+^E-cad^+^; Fig. 5b). Treatment with ICG-001 dramatically reduced the abundance of this stem-like cell population (Fig. 5c). Further, immunofluorescence staining for β-catenin of FACS-isolated CD29^+^CD24^high^CD133^+^E-cad^+^ cells from those tumors confirmed that ICG-001 treatment promoted membrane localization of β-catenin (Fig. 5d). Thus, ICG-001 inhibited aggressive phenotypes of TC-1 cells in a manner similar to its effects on OSCC cells and tumors.

### ICG-001 selectively targets a stem cell-like tumor subpopulation in vivo

The transcriptional activity of β-catenin has been shown to promote the expansion of subpopulations of cells with immature stem-cell-like phenotypes [20, 43]. These cells exhibit the ability to self-renew are capable of seeding tumors at low numbers in mouse xenografts and driving tumor progression to metastasis. In particular, a subset of β-catenin-positive cells from HNSCC, expressing the tumor stem cell marker CD44, were also shown to co-express CD24 and CD29 and to promote non-adherent tumor sphere growth and seed tumors at low numbers in mice [20]. Thus, to assess if ICG-001 inhibited the ability of CD44^+^CD24^high^CD29^+^ cells to drive rapid OSCC tumor growth and metastases, we adapted an embryonic zebrafish xenograft model to screen for aggressive human stem cell-like OSCC cells. Due to its small size, transparency, and close homology with its human counterpart, the zebrafish xenograft model possesses unique advantages enabling the rapid screens of aggressive human OSCC stem-like cells [48].

**Fig. 6:**
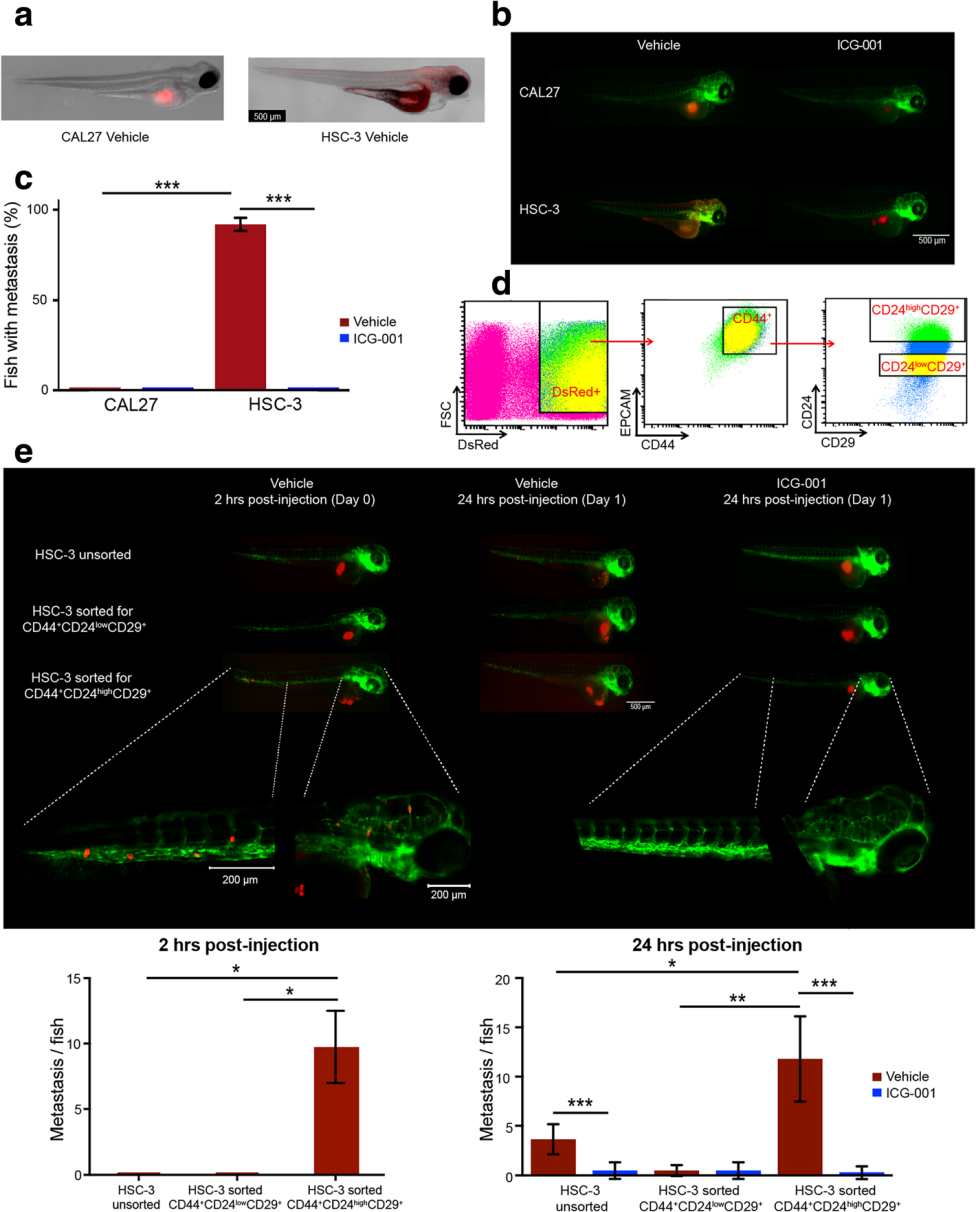
Zebrafish embryos facilitate rapid detection of an aggressive stem-like subpopulation of OSCC cells and of the effects of ICG-001 inhibition. **a** HSC-3 and CAL27 cells were transduced with a CellTracker™, and 100–200 cells in ~ 1 nL were injected directly into the periviteline space of the embryo. After 24 h, CAL27 cells formed tumors that did not metastasize, whereas HSC-3 cells produced larger tumors that metastasized to the tail and to the craniofacial region. **b** Immunofluorescence imaging of CellTracker™-labeled CAL27 and HSC-3 cells revealed that treatment with ICG-001 inhibited CAL27 cell-driven tumor growth, as well as growth and metastasis of HSC-3-derived tumors. **c** Percent of injected fish exhibiting metastasis was quantified and displayed as the mean (± S.D.) per cell type and per treatment group. Majority of vehicle-treated HSC-3-driven tumors (*n* = 86) metastasized relative to vehicle-treated CAL27-driven tumors (*n* = 68). ICG-001 treatment prior to injection resulted in a significant reduction in percent fish displaying HSC-3-driven metastasis (*n* = 104). **d** Identification of subpopulations of RFP-transduced HSC-3 cells with primitive antigenic cell markers CD44^+^CD24^high^CD29^+^ by flow cytometry. **e** Subpopulations of CD44^+^CD24^high^CD29^+^ and CD44^+^CD24^low^CD29^+^ cells were isolated by FACS, grown in culture for 12 h followed by treatment with either DMSO (vehicle) control or ICG-001 for 24 h. Unsorted HSC-3 cells were used as control. Immunofluorescence imaging of tumor xenografts derived from unsorted RFP HSC-3 cells (*n* = 3), RFP CD44^+^CD24^low^CD29^+^ cells (*n* = 3), or CD44^+^CD24^high^CD29^+^ cells (*n* = 3) revealed that only CD44^+^CD24^high^CD29^+^ cells formed tumors that metastasized rapidly within 2 h to the tail and craniofacial regions (left panel). After 24 h, metastases were detected from tumors derived from CD44^+^CD24^high^CD29^+^ (*n* = 5) as well as unsorted cells (*n* = 6), and to a much lesser extent, from CD44^+^CD24^low^CD29^+^ cells (*n* = 6; middle panel). We note that a substantial population of zebrafish injected with CD44^+^CD24^high^CD29^+^ cells died within the 24 h (middle panel). Treatment of CD44^+^CD24^high^CD29^+^ cells (*n* = 11) as well as unsorted cells (*n* = 6) with ICG-001 inhibited tumor growth and significantly abrogated metastases (right panel). In each case, metastasis was quantified as number of metastasis per fish (see “Methods”), and displayed as the mean (± S.D.). *P < 0.01; **P < 0.001; ***P < 0.0001; unpaired *t* test

We first examined the behavior of CAL27 and HSC-3 cells in 2-day post-fertilization (2-dpf) zebrafish, which lack an adaptive immune system. CAL27 and HSC-3 cells were labeled with a fluorescent tracker dye and 100–200 cells were injected into zebrafish embryos as described (see “Methods”). The behavior of cell tracker dye-stained OSCC cells in zebrafish closely mimicked our orthotopic mouse models in their ability to grow tumors without and with metastatic properties (*P* < 00001; two-tailed *t* test), respectively (Fig. 6a–c). Additionally, treatment with ICG-001 significantly reduced metastasis in zebrafish tumors induced by HSC-3 cells (*P <* 0.0001, two-tailed *t* test; Fig. 6b–c), as it did in murine models.

Since it has been shown that a specific subpopulation of aggressive tumor cells is associated with stem cell-like properties [20], we assessed CD24, CD29, and CD44 expression in HSC-3 cells by flow cytometry. HSC-3 cells, transduced with lentiviral red fluorescent protein (RFP), displayed high levels of CD44 and CD29 expression but varying levels of CD24 expression. To determine the role of β-catenin on aggressive behavior of OSCC cell subpopulations, we used fluorescence-activated cell sorting (FACS) to isolate CD44^+^CD24^low^CD29^+^ and CD44^+^CD24^high^CD29^+^ cells from RFP-expressing HSC-3 cells (Fig. 6d). After sorting, CD44^+^CD24^low^CD29^+^, CD44^+^CD24^high^CD29^+^, and unsorted HSC-3 cells were cultured for 12 h and then treated with either vehicle control (DMSO) or 10 μM ICG-001 for 24 h. Remarkably, untreated CD44^+^CD24^high^CD29^+^ cells showed rapid growth and metastasis within the first 2 h following injection. This was in sharp contrast to CD44^+^CD24^low^CD29^+^ cells (*P* = 0.002*,* two-tailed *t* test) and unsorted cells (*P* = 0.002, two-tailed *t* test), which produced smaller tumors with no detectable metastases until 24 h post-transplantation (Fig. 6e). After 24 h, the CD44^+^CD24^high^CD29^+^ cells exhibited more extensive metastases compared to both the unsorted (*P* = 0.001, two-tailed *t* test) and CD44^+^CD24^low^CD29^+^ groups (*P* = 0.0001, two-tailed *t* test). Notably, ICG-001 treatment significantly reduced metastasis of CD44^+^CD24^high^CD29^+^ (*P* < 0.0001, two-tailed *t* test) and unsorted cells (*P* < 0.0001, two-tailed *t* test; Fig. 6e), consistent with its proposed role in targeting β-catenin activity in immature cancer cells [20, 24]. Thus, inhibition of β-catenin activity by treatment with ICG-001 virtually eliminated metastasis of CD44^+^CD24^high^CD29^+^ cells in zebrafish embryos, confirming that β-catenin/CBP-mediated transcriptional activity was critical for the maintenance of aggressive human OSCC CSCs in vivo.

### ICG-001 treatment signature is associated with tumor progression and patient survival in primary human OSCC

**Fig. 7:**
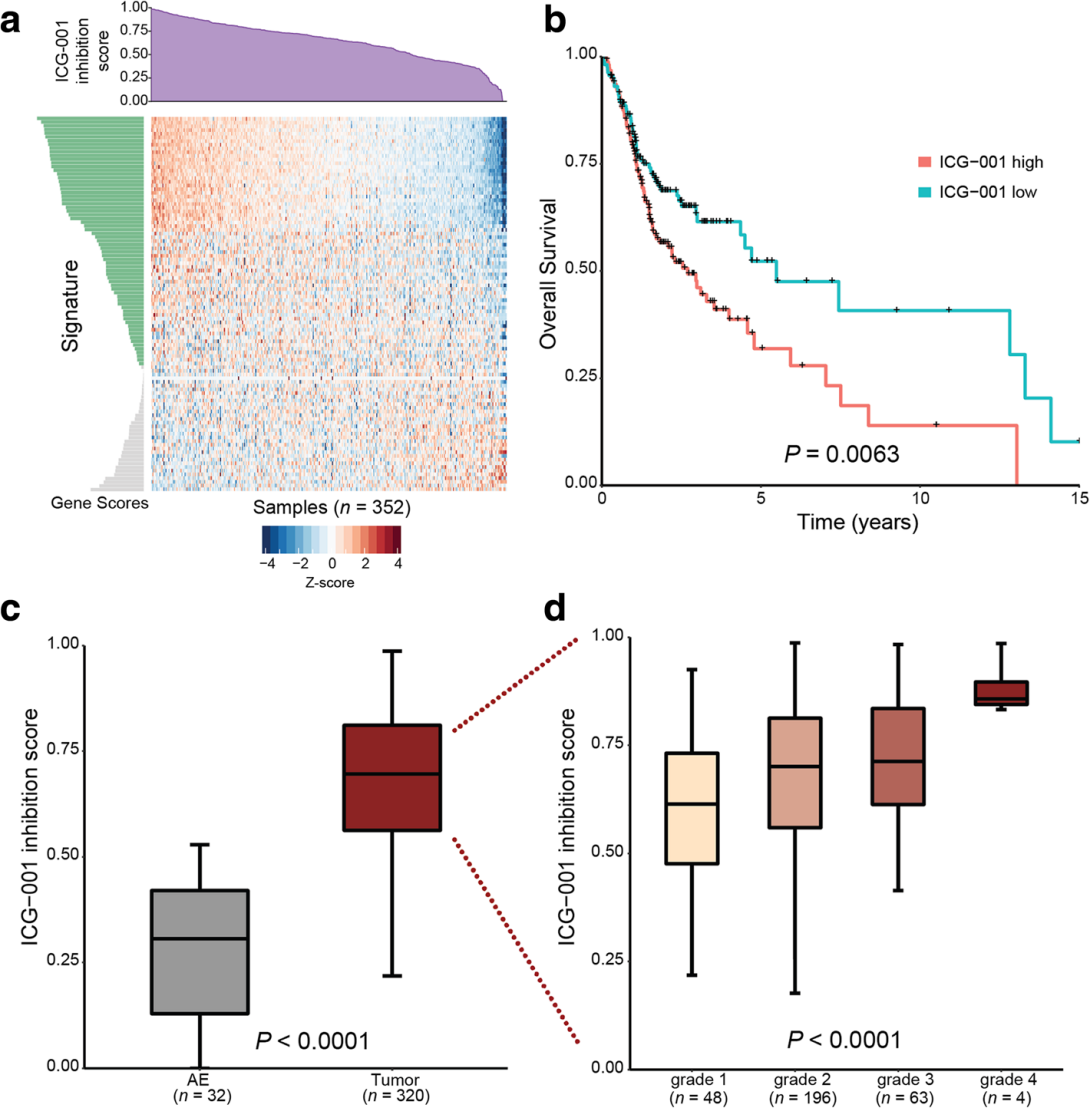
Transcriptional ICG-001 inhibition signature is associated with clinical outcomes in primary human OSCCs. **a** Stratification of human OSCC samples using the ICG-001 inhibition gene expression signature from HNSCC cells. The TCGA OSCC RNASeq dataset (*n* = 352) was projected onto a core set of genes downregulated by ICG-001 in both HSC-3 and CAL27 cells (*n* = 104 genes) using ASSIGN. Heatmap of the signature expression profile (displayed as row *z* scores of log_2_ expression values per gene) is shown, with the estimated ICG-001 inhibition scores displayed in the barplot above (purple). **b** Overall patient survival curves with respect to ICG-001 inhibition score. OSCC patients with survival information (*n* = 318) were first divided into two groups: ICG-001 high and low, *n* = 159 per group, based on the median ICG-001 inhibition scores and compared for their overall survival outcomes. Samples with higher ICG-001 inhibition scores had a lower overall survival, with median survival estimates of 2.7 and 5.48 years in the high and low groups, respectively. Statistical significance of survival difference between groups was determined using a log-rank test **c** ICG-001 inhibition scores with respect to OSCC tumor status. OSCC samples (*n* = 320) have significantly higher ICG-001 inhibition scores than adjacent normal epithelia (AE) (*n* = 32). Statistical significance was determined using an unpaired *t* test comparing the two groups. **d** OSCC samples with known tumor grade status (*n* = 311) were used to analyze the association of ICG-001 inhibition scores and tumor progression. A significant positive association was observed between ICG-001 inhibition scores and increasing tumor grade (see “Methods”)

To align the activity of genes repressed by ICG-001 with clinically aggressive OSCC, we assessed the derived ICG-001-inhibited gene signature in primary human OSCC samples by querying RNA-sequencing (RNASeq) data from the cancer genome atlas (TCGA). Specifically, we projected the TCGA OSCC data (*n* = 352 samples) in the space of the core ICG-001 inhibition signature described earlier, thus capturing shared inhibitory activity over broader OSCC phenotypes (see “Methods”). TCGA samples were scored based on the coordinated expression of the signature genes, with a high (low) score corresponding to coordinated up(down) regulation of the signature genes, which in turn, reflected the level of potential ICG-001 inhibition (i.e., the ICG-001 inhibition score) per sample (Fig. 7a). The expectation was that samples with high inhibition score (i.e., with coordinated upregulation of the signature genes) would be more responsive to ICG-001 treatment. Thus, we tested whether ICG-001 inhibition scores were predictive of patient outcome, as measured by the overall survival (OS) estimates for OSCC patients (*n* = 318). Comparison of the survival curves corresponding to patients with high (> median) or low (≤ median) ICG-001 inhibition scores showed a significantly lower OS in the former group (Fig. 7b; *P* = 0.0063, log-rank test), with median survival estimates of 2.7 and 5.48 years in the high and low groups, respectively. This confirmed that the ICG-001 inhibition signature was predictive of patient prognosis in OSCC and suggested that patients with high inhibition scores were likely to be responsive to the treatment targeting β-catenin/CBP activity. Furthermore, comparison of the ICG-001 inhibition scores in OSCCs (*n* = 320) versus normal adjacent epithelial (AE) samples (*n* = 32) yielded significantly higher scores in OSCCs than in AE’s (Fig. 7c; *P* < 0.0001, two-tailed *t* test), thus showing that ICG-001 inhibition was associated with tumor status. We then assessed samples with known tumor grade information (*n* = 311) to determine the association between ICG-001 inhibition scores and tumor progression. Remarkably, ICG-001 inhibition scores were found to significantly increase with advanced tumor grade (Fig. 7d; combined *P* < 0.0001), suggesting a correlation with tumor progression. Taken together, these results show that higher inhibition scores in primary tissues are associated with tumor status and positively associated with tumor progression and suggest that more aggressive/advanced tumors would be more responsive/susceptible to inhibition by ICG-001.

## Discussion

Identifying oncogenic activities that drive HNSCC and represent bona fide therapeutic targets is paramount to finding effective treatments for patients with this malignancy. The challenge to identifying new biomarkers and targets for therapeutic interventions is exacerbated by the intra-tumoral heterogeneity of HNSCC and diversity of anatomical sites and subsites, many with different underlying biology. Emerging computational methodologies coupled with genomic analyses have provided powerful tools for the identification of molecular circuitries, new druggable targets, and biomarkers relevant to disease progression and treatment response. We have reported previously that integrating in vitro treatment signatures with high-throughput data from consortia such as the TCGA network facilitated decoding their significance in OSCC when coupled with functional analyses [40, 49]. Here, we applied computational approaches to interrogate gene expression signatures in response to the disruption of β-catenin-CBP interaction with a small molecule antagonist, ICG-001, in OSCC cell lines and to align them with patho-clinical features of human OSCC tumors. We further validate anti-tumor effects of ICG-001 in vivo using tumor xenografts in zebrafish and mouse models and show that this antagonist of β-catenin/CBP activity specifically targets subpopulations of cells expressing stem-like cell surface markers. Our studies highlight the significance of β-catenin/CBP signaling in the pathobiology of OSCC and suggest that inhibition of β-catenin-CBP activity with ICG-001 promotes epithelial differentiation program. By investigating TCGA OSCCs, we demonstrate that gene expression changes resulting from ICG-001 inhibition are associated with higher tumor grade and lower overall survival, suggesting that transcriptional signature associated with the disruption of β-catenin-CBP interaction has prognostic value. Our work provides new evidence for the feasibility of using inhibitors of β-catenin/CBP activity for the treatment of head and neck cancer patients.

One important outcome of this study is the identification of a shared transcriptional program associated with the inhibition of β-catenin/CBP with ICG-001 in OSCC. Using microarray data from CCLE, we show that the core ICG-001 inhibition signature derived from OSCC cells can be used to predict sensitivity of additional cell lines to ICG-001 based on coordinated expression of these core genes. To this end, we show that pharyngeal SCC FaDu cells are indeed sensitive to ICG-001, although not all gene products we show to be repressed by this antagonist in OSCC HSC-3 cells are altered in FaDu cells, suggesting cell- and tumor site-specific differences. Nonetheless, similar to OSCC CAL27 and HSC-3 cells, the ICG-001 treatment led to downregulation of the CBP steady-state levels in FaDu cells. The acetyltransferase activity of CBP promotes an open chromatin structure by acetylation of histone and non-histone proteins [50], and when complexed with β-catenin, it induces the expression of stem-like, proliferation and survival genes [20]. Thus, inhibition of β-catenin/CBP signaling by ICG-001 would be expected to counteract epigenetic changes aligned with immature states and promote a chromatin structure associated with cellular differentiation. Accordingly, our genomic and biochemical analyses show that in OSCC cell lines, ICG-001 suppresses stem cell-like and survival markers and enhances the expression of epithelial differentiation genes. Besides *CLDN1*, *CLDN4*, *CDH4*, and *ICAM1*, ICG-001 induced the expression of additional genes associated with epithelial differentiation, including *SLP1*, *KLK7*, *KLK10*, and *KRT7* in HSC-3 cells [51]. In mouse models of tongue OSCC, ICG-001 inhibited invasive tumor growth and promoted a capsular phenotype along with cellular characteristics consistent with enhanced intercellular adhesion that accompanies cellular differentiation. Further, ICG-001 abrogated the ability of subpopulations of human OSCC cells with stem cell-like properties to induce rapid tumor formation and metastases in zebrafish. Notably, the ICG-001 treatment-associated inhibition signature stratified primary OSCCs based on tumor grade and overall survival. We thus hypothesize that the ICG-001 inhibition signature represents a transcriptional program of patients more likely to respond to the inhibitor. Although current diagnoses and treatment decisions for patients with HNSCC rely on tumor stage rather than grade, recent single-cell RNA–sequencing-based characterization of human HNSCCs revealed a new partial epithelial-to-mesenchymal transition program associated with loco-regional invasion and metastases, as well as higher tumor grade but not tumor size [51]. Further, epithelial differentiation was negatively associated with metastases. Therefore, our observation that ICG-001 inhibition signatures track with tumor grade may prove important to future therapies.

Another novel finding from this study is that disruption of the β-catenin-CBP interaction with ICG-001 in OSCC cell lines destabilizes CBP without greatly affecting E-cadherin or β-catenin transcript or protein levels. Previous analyses mapped the binding of ICG-001 to the 111N-terminal amino acids of CBP, required for the interaction with the C-terminal domain of β-catenin to induce TCF/LEF-mediated expression of target genes [25]. Given that CBP is involved in the co-activation of different transcription factors, these mapping studies revealed a selectivity of ICG-001 for a fraction of genes impacted by the interaction of CBP with β-catenin. The dramatic inhibition of CBP levels by ICG-001 in OSCC and pharyngeal SCC cell lines suggests that these cells depend on β-catenin-CBP signaling for proliferation and survival. CBP has been suggested to be rate limiting in its transcriptional activities [25], although how the steady-state levels of CBP align with aggressive HNSCC cell phenotypes is unclear. While further investigation is needed to determine the exact mechanism underlying downregulation of nuclear CBP protein levels by ICG-001, our studies suggest that the binding of its N-terminal region to β-catenin provides protection from degradation.

In addition to directly inhibiting β-catenin/CBP signaling, our studies show that ICG-001 affects activities of other pathways that converge on the Wnt/β-catenin pathway. We have previously demonstrated that the transcriptional activities of two Hippo pathway effectors, YAP and its paralog TAZ, track with OSCC development and progression to advanced state [49]. GSEA of the YAP/TAZ-associated signature revealed a significant positive correlation between ICG-001 treatment and YAP/TAZ knockdown in HSC-3 cells (Additional file 1: Figure S8b; FDR *q* < 0.001), suggesting potential convergence between YAP/TAZ and CBP and/or β-catenin in OSCC [52]. Moreover, in our prior studies, we showed that Wnt/β-catenin signaling contributed to the induction of the Wnt/Planar Cell Polarity (Wnt/PCP) pathway in oral cancer [32]. Thus, it is likely that ICG-001 also interferes with the Wnt/PCP pathway along with its associated oncogenic features.

The effects of ICG-001 inhibition in OSCC resemble those initially described in colorectal cancer (CRC) with some exceptions. Similar to OSCC, ICG-001 inhibited β-catenin/TCF signaling and reduced CRC cell growth in vitro and in nude mouse xenografts in vivo. Furthermore, in CRC cell lines, ICG-001 inhibited the expression of the *BIRC5* gene product, survivin, coincident with upregulation of caspase 3 activity [25]. While *BIRC5* transcript and survivin protein levels were inhibited by ICG-001 in OSCC cell lines, we did not detect apoptosis or increased caspase 3 levels, indicating that in OSCC, ICG-001 was cytostatic. Also, analogous to OSCC, ICG-001 did not impact β-catenin levels in CRC cell lines but, unlike OSCC, ICG-001 did not inhibit CBP levels, indicating tumor tissue-specific differences in response to this inhibitor.

Current FDA-approved targeted therapies for HNSCC are limited to cetuximab, a monoclonal antibody directed at the epidermal growth factor receptor (EGFR), and pembrolizumab and nivolumab, anti-programmed cell death-1 (PD-1) targeted immunotherapies [53]. These therapies have modest clinical response rates, and there are few biomarkers available to predict response. Our genomic and functional analyses provide evidence that β-catenin/CBP activity plays a pivotal role in the pathobiology of OSCC and is associated with poor clinical outcomes. Given that no inhibitors of β-catenin activity have entered clinical trials for the treatment of head and neck cancer, our studies may facilitate the development of therapies targeting β-catenin/CBP activity in human patients.

## Conclusions

Although HNSCC is a devastating disease that continues to mount a major global health problem [13, 14], current clinical management of this malignancy remains limited by factors such as treatment-associated morbidity, resistance to conventional therapy, and disease persistence/recurrence [54]. Decoding the underlying molecular mechanisms involved in HNSCC development and progression to aggressive disease is critical to the discovery of new druggable targets and effective treatment paradigms. Using cellular and animal models, as well as human data, we highlight the contribution of the β-catenin/CBP signaling axis to HNSCC and provide evidence that targeting this signaling activity may be a useful strategy to intercept this malignancy.

## Additional files

Additional file 1: Document describing Supplemental Materials and Methods, also including **Table S1.** and **Figures S1-S8.** (DOCX 2768 kb)

Additional file 2: Tabular spreadsheet file containing both table of differential gene expression results comparing ICG-001 treatment (*n* = 3) versus DMSO (vehicle) control (*n* = 3) in HSC-3 and CAL27 OSCC cell lines, as well as the ICG-001 treatment gene expression signatures derived thereof (see manuscript “Methods”). (XLSX 4246 kb)

Additional file 3: Tabular ranked gene list file (viewable in Excel) pertaining to differential gene expression testing comparing siRNA-mediated knockdown of β-catenin versus scrambled siRNA control in HSC-3 cells used for GSEA. Fields indicate gene symbol and *t* test statistic of differential expression, respectively, used as input to the pre-ranked GSEA tool. (TXT 289 kb)

## Abbreviations

ASSIGN: Adaptive Signature Selection and Integration
CBP: cAMP response element-binding (CREB)-binding protein
CCLE: Cancer Cell Line Encyclopedia
CSCs: Cancer stem cells
FDR: False discovery rate
GSEA: Gene Set Enrichment Analysis
HNSCC: Head and neck squamous cell carcinoma
OS: Overall survival
OSCC: Oral squamous cell carcinoma
RNASeq: RNA-sequencing
SCC: Squamous cell carcinoma
TCGA: The Cancer Genome Atlas
UAT: Upper aerodigestive tract
