# Supplementary material for: Functional and genomic analyses reveal therapeutic potential of targeting β-catenin/CBP activity in head and neck cancer
Source: Genome Med. 2018 Jul 20;10:54. doi: 10.1186/s13073-018-0569-7 (PMC6053793; doi:10.1186/s13073-018-0569-7)
Supplement: Supplementary file 1 — Document describing Supplemental Materials and Methods, also including Table S1. and Figures S1-S8. (DOCX 2768 kb) [file 13073_2018_569_MOESM1_ESM.docx]

Supplemental Information pertaining to *Kartha et al. Functional and genomic analyses reveal therapeutic potential of targeting β-catenin/CBP activity in head and neck cancer*

# Supplemental Materials and Methods

## TOPflash β-catenin activity assay

HSC-3 cells were plated 1 x 10^5^ cells per well in 24-well plates in complete media (DMEM supplemented with 10% fetal bovine serum) and allowed to adhere overnight. Cells were then transfected, using 2 ul Lipofectamine 2000 (Thermo Fisher Scientific) with 0.1 ug of pRLnull and either 0.9 ug of M50 Super 8x TOPflash or M51 Super 8x FOPflash (TOPflash mutant) reporter construct (gifts from Randall Moon (Addgene plasmids #12456 and #12457) per well [1] using manufacturer’s standard protocol and as previously reported [2]. Following transfection, cells were washed once in complete media and then treated with complete media containing with vehicle or 10µM ICG-001 in triplicate. Vehicle (DMSO) concentration was uniform at 0.2% for all treatment conditions. Following 48 hours of treatment, cells were washed once with PBS, and luciferase activities determined using the Dual-Luciferase® Reporter Assay System (Promega) according to the manufacturer protocol. TOP/FOPflash activity was expressed relative to the transfection control as relative luciferase units (RLUs).

## Immunofluorescence imaging

For immunofluorescence analysis of β-catenin and F-actin, CAL27 cells (1.x10^4^) were seeded in 6-well chamber slides in the presence of either 1% DMSO or 10 μM ICG-001. Cells were grown to 70% confluence on Nunc Lab-Tek II Chamber Slide (Thermo Fisher Scientific), fixed in 3.7% paraformaldehyde, permeabilized with 0.1% Triton X-100, blocked with 10% goat serum, and incubated with primary antibodies to β-catenin (Abcam, rabbit polyclonal AB) followed by secondary antibody, goat anti-rabbit conjugated with Alexa Fluor 488 (Jackson ImmunoResearch). Sections were counterstained for F-actin with rhodamine-conjugated phalloidin and for nuclei with 4'6-diamidino-2-phenylindole, dihydrochloride (DAPI) (Molecular Probes), mounted in ProLong Gold Antifade, (Molecular Probes) and images were analyzed with a Zeiss LSM 710-Live Duo Scan confocal microscope. To insure valid comparison of fluorescence intensities between samples, settings were fixed to the most highly stained sample and all other images were acquired at those settings.

## RT-qPCR

Real-time quantitative-PCR (RT-qPCR) was used to validate select genes’ relative expression comparing ICG-001 and DMSO (Vehicle) treatment in CAL27 and HSC-3 cells. Total RNAs were extracted from CAL27, HSC-3 cells using miRNeasy Micro RNA isolation kit (Qiagen, Cat # 217084). cDNA synthesis was performed using 1µg RNA and SuperScript III First –Strand cDNA synthesis kit (Invitrogen Cat. # 18080-051) according to manufacturer’s protocol. RT-qPCR was performed using SYBR Green PCR master mix (Applied Biosystems, Cat. # 4309155), and measured on StepOne Plus Real-Tyme PCR System machine (Applied Biosystems). Transcript levels were analyzed using the ∆∆C_t_ method and normalized to GAPDH. Statistical analysis was performed using real time PCR from three independent RNA preparations, with each experiment repeated 3 times (*n* = 9) using a two-tailed Student’s *t-* test. Primer sequences for selected genes are indicated in Table S1.

## FaDu cell culture

The human hypo-pharyngeal carcinoma FaDu cell line was obtained from ATCC. FaDu cells were cultured as a monolayer in MEM (Minimum Essential Medium Eagle) containing 10% fetal calf serum, 100 U/ml penicillin, 100 mg streptomycin, 1mM sodium pyruvate and 2 mM L- glutamin at 37^0^ C in a humidified atmosphere composed of 95% air and 5% CO_2_. EC_50_ concentration for sensitivity to ICG-001 was determined as for OSCC cell lines assessed (see manuscript methods). Cells were treated with either DMSO (vehicle) or ICG-001 as for OSCC cell lines (see manuscript methods), and were assessed for protein marker quantification using immunoblot analysis (see below).

## Immunoblot analysis

Samples (40 μg of total protein) were analyzed by immunoblot according to a standard protocol as previously described [3] using primary antibodies to E-cadherin, 1:1000 (BD Bioscience); CBP, 1:1000 (Cell Signaling); β-catenin, 1:1000 (Sigma); GAPDH, 0.2 μg/ml (Sigma); Keratin 14, 1:1000 (Abcam); HELLS, 1:500 (Invitrogen); Survivin, 1:500 (Invitrogen); Claudin1, 1:25 (Abcam). Protein expression was detected by chemiluminescence (SuperSignal West Pico Plus, Pierce) and quantified with either a biomolecular imager ImageQuant^TM^ LAS 4000 or NIH software ImageJ.

## Nuclear and cytoplasmic fractionation

HSC-3 cells were grown in DMEM to 50 - 60% confluence in the presence of either 1% DMSO or 10 μM ICG-001/0.1% DMSO, as described in the manuscript methods and [3]. For nuclear and cytoplasmic fractionation, 2 x10^6^ cells per condition were harvested and processed for nuclear and cytoplasmic extraction using NE-PER Extraction Reagents per manufacturer’s instruction (Thermo Scientific, #78835). In addition, TCLs were prepared from same numbers of cells under identical conditions. For immunoblot analyses, 25 μg of total protein from TCL, cytoplasmic and nuclear fractions were analyzed on 7.5% Mini-PROTEAN® TGX™ Precast Protein Gels (Bio-Rad, #4561086) and processed for β-catenin and CBP expression using anti-β-catenin and anti-CBP antibodies, as described above.

## Transcriptional profiling of β-catenin knockdown

Three separate wells (triplicate) from 6 well plates were seeded by HSC-3 cells (around 5x10^4^/ well). After 48 hours, cells were transfected with 25 nM SMART pool ON-TARGET plus CTNNB1 siRNA (L-003482-00-0005, Dharmacon) by reverse transfection. ON-TARGET plus non-targeting Pool 25nM (D-001810-10-05, Dharmacon) was used for negative control. The cells were trypsinized and harvested after 48 hours for RNA purification for qPCR of CTNNB1 and genome-wide microarray gene expression profiling. CTNNB1 qPCR estimates were obtained by normalizing the obtained Ct with a housekeeping gene GAPDH. Expression estimates were then compared with cells treated with non-targeting siRNA (∆∆C_t_), confirming 90% knockdown efficiency. RNA purification, microarray transcriptional profiling and differential expression of β-catenin knockdown with control was performed as described for ICG-001 treatment (see manuscript Materials and Methods).

## Gene Set Enrichment Analysis (GSEA)

GSEA v2.2.1 desktop software was used to perform enrichment analysis on the derived ICG-001 treatment profiles [4]. Pre-ranked GSEA was run for the β-catenin and YAP/TAZ knockdown (KD) in HSC-3 cells, using the *t*-statistic of microarray differential expression results comparing siRNA to CTNNB1 or YAP/TAZ versus scrambled siRNA control conditions for β-catenin and YAP/TAZ KD, respectively, as the continuous ranking variable (*n*=23,744 and 23,466 ranked reference genes for siCTNNB1 and siYAP/TAZ, respectively). Gene sets pertaining to ICG-001 treatment in HSC-3 cells were derived as described in the manuscript Materials and Methods section, and were queried for enrichment within the ranked β-catenin and YAP/TAZ KD reference gene lists. YAP/TAZ KD microarray data was generated and analyzed as previously described [5]. All default parameters of the GSEA software were used.

# Supplemental Tables

| **Target** | **Forward primer sequence** | **Reverse primer sequence** |
| --- | --- | --- |
| LEF1 | 5’ CAGGAGCCCTACCACGACAA 3’ | 5’ CCTCCATCTGGATGCTTTCC 3’ |
| SKP2 | 5’ AAAGAGGAGCCCGACAGTGA 3’ | 5’ GGGTGGCCCAGGTTTGAG 3’ |
| BIRC5 | 5’ GCCAAGAACAAAATTGCAAAGG 3’ | 5’ TTTCTCCGCAGTTTCCTCAAA 3’ |
| CCNE2 | 5’ CATGGTGTTCAACCTGTGCTCTA 3’ | 5’ GGAGCCACAGCATTTATCTTGTT 3’ |
| CDH4 | 5’ CAGGACAGTGAGAGGGTCTGTGT 3’ | 5’ GAGAATGCACCCTCTGAGTTTTC 3’ |
| CLDN1 | 5’ CTGGGAGGTGCCCTACTTTG 3’ | 5’ CTTGGTGTTGGGTAAGAGGTTGT 3’ |
| HELLS | 5’ AAGGGACTTTGCAGATTCATCCT 3’ | 5’ CATTTCGGTCTCTCATGGCTATT 3’ |
| NR5A2 | 5’ CCGAGCCAATGGACTTAAGCT 3’ | 5’ TGGTCAGGTCAGAGGGCATAG 3’ |
| GAPDH | 5’ GGTCTCCTCTGACTTCAACA 3’ | 5’ AGCCAAATTCGTTGTCATAC 3’ |

**Table S1**. Primer sequences used for qRT-PCR validation. All sequences for target genes correspond to Human orthologues.

# Supplemental Figures

**
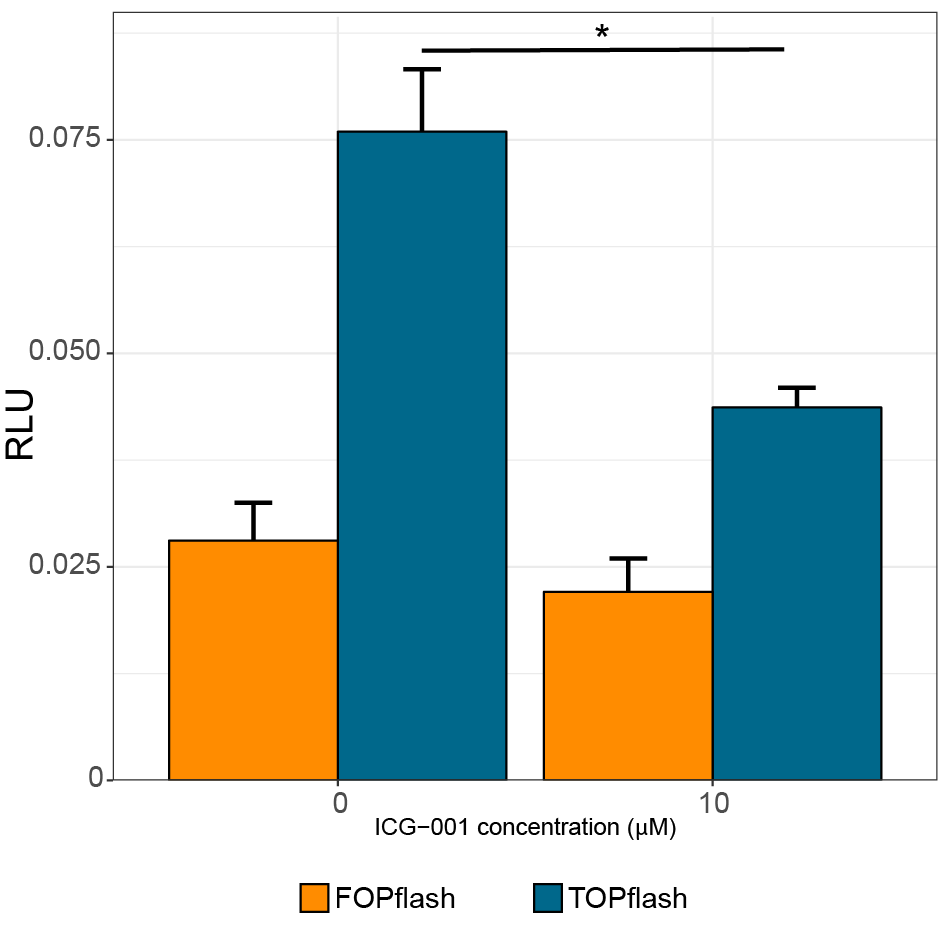
**

**Figure S1.** TOPflash assay confirming the inhibitory impact of ICG-001 on β-catenin-mediated transcriptional activity in HSC-3 cells. HSC-3 cells were treated with either no (DMSO vehicle) or 10 µM ICG-001 for 48 hours, following which luciferase activity was determined. TOP/FOPflash activity was expressed relative to the transfection control as relative luciferase units (RLUs). Relative luciferase activity is displayed as mean + S.D RLUs (*n*=3). * *P* < 0.005 Student’s *t*-test.


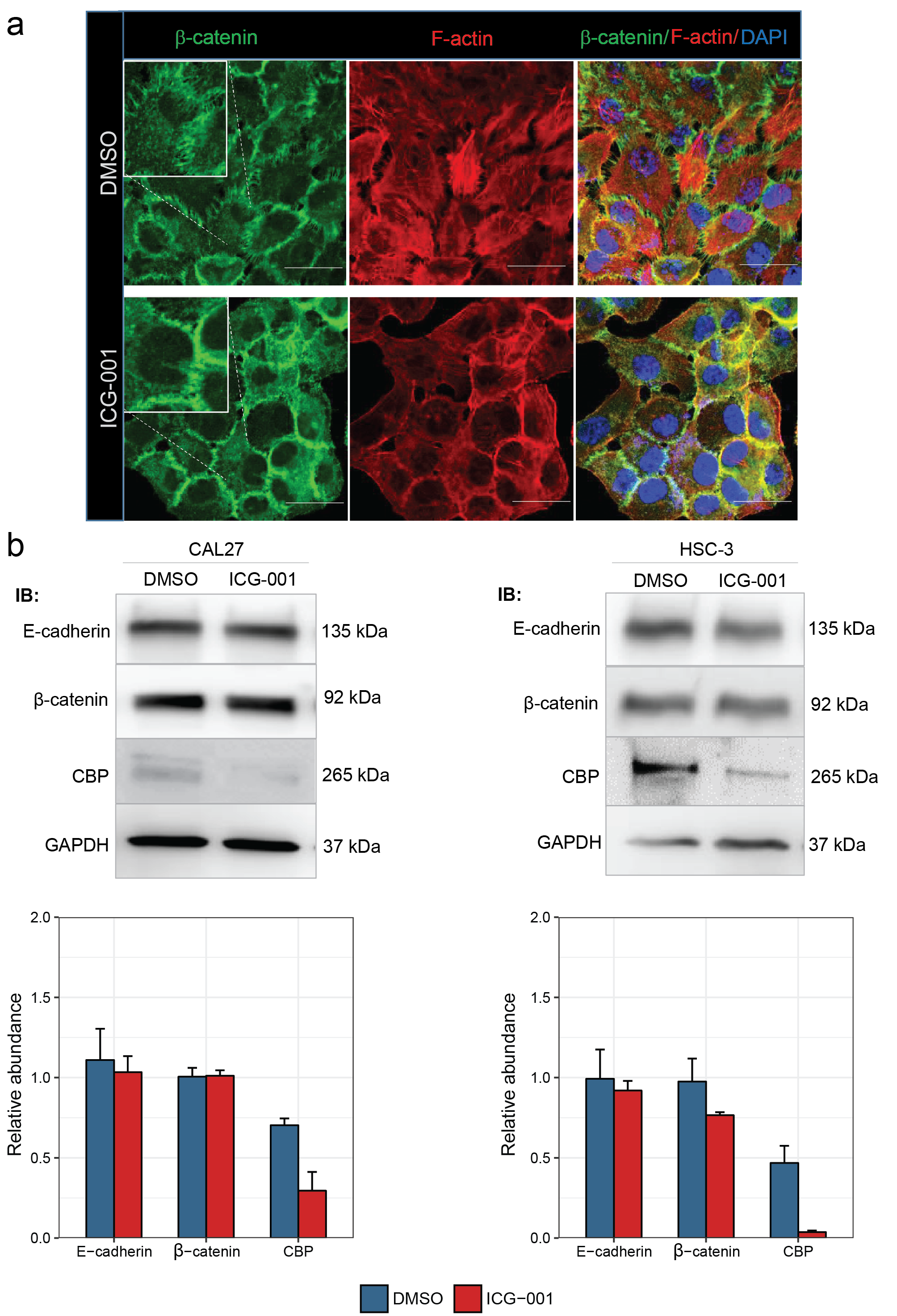


**Figure S2**. Inhibition of β-catenin-CBP interaction with ICG-001 promotes junctional localization of β-catenin and epithelial morphology, while diminishing CBP protein levels in OSCC cells **a**. Immunofluorescence imaging of β-catenin and filamentous actin (F-actin) in DMSO-treated CAL27 cells displays a broad localization of β-catenin at membrane extensions between cells. Following the ICG-001 treatment, β-catenin displays enriched localization at membrane domains coincident with the loss of stress fibers and enhanced epithelial morphology. Merged images show increased co-localization of β-catenin and F-actin in ICG-001-treated cells suggesting enhanced junctional organization in these cells. Size bars, 10 μm. **b**. ICG-001 down-regulates CBP protein levels in CAL27 and HSC-3 cells. Immunoblots of E-cadherin, β-catenin and CBP in CAL27 and HSC-3 cells treated with either vehicle control (DMSO) or ICG-00, with GAPDH as a loading control (top). Bar plot of relative abundances are shown below as mean (+s.d.) GAPDH-normalized values (*n*=2 per treatment condition). IB: immunoblot.

**
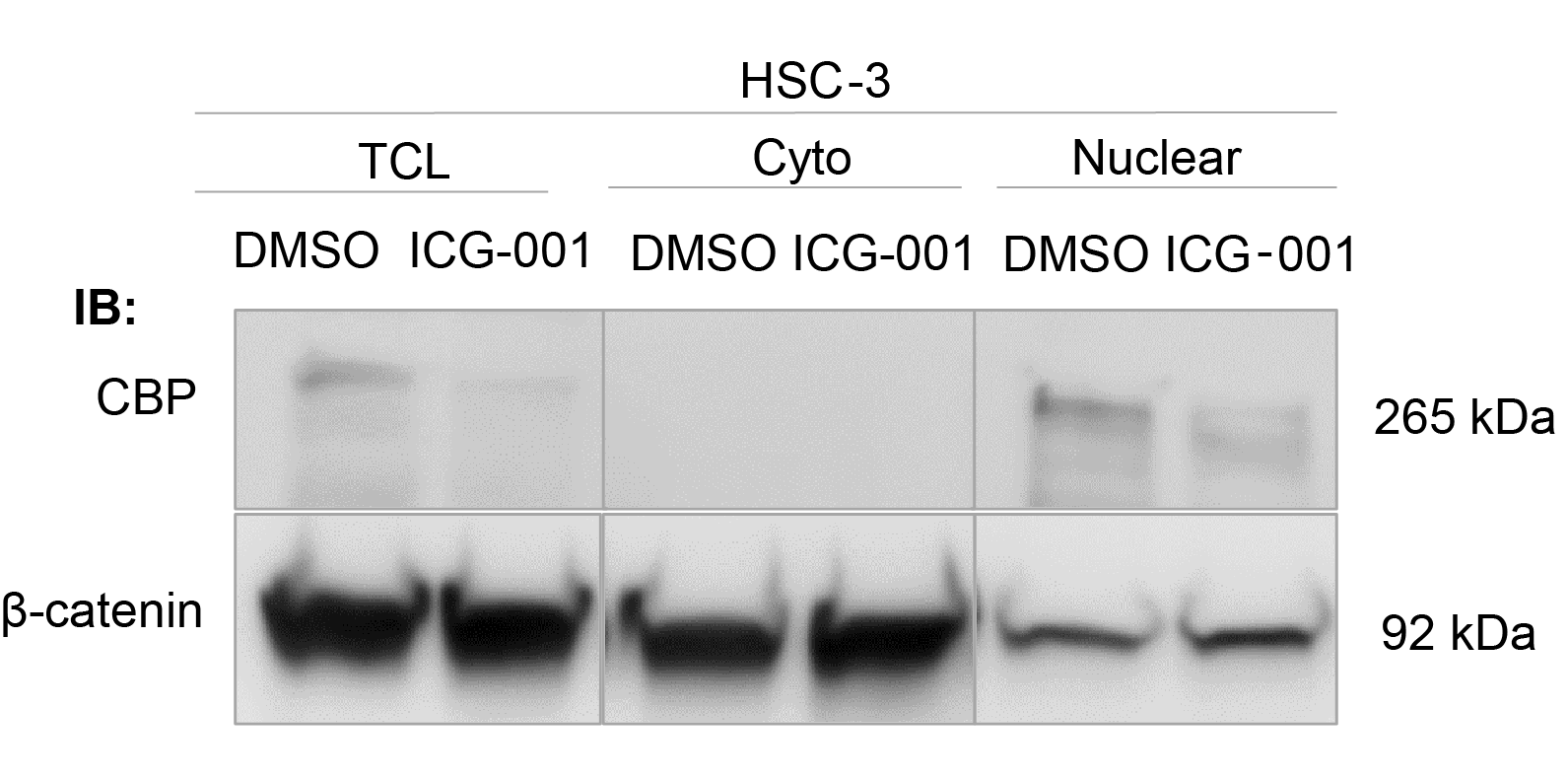
**

**Figure S3**. ICG-001 specifically alters nuclear CBP abundance in HSC-3 cells. CBP and β-catenin protein levels were quantified using total cell lysate (TCL), and cytoplasmic (Cyto) or nuclear fractions isolated from HSC-3 cells, subjected to treatment with either DMSO (control) or ICG-001 and then quantified using immunoblot assay. No change in total or fractionated β-catenin level is observed, while nuclear and TCL CBP is down-regulated at the protein level upon ICG-001 treatment. IB: immunoblot.


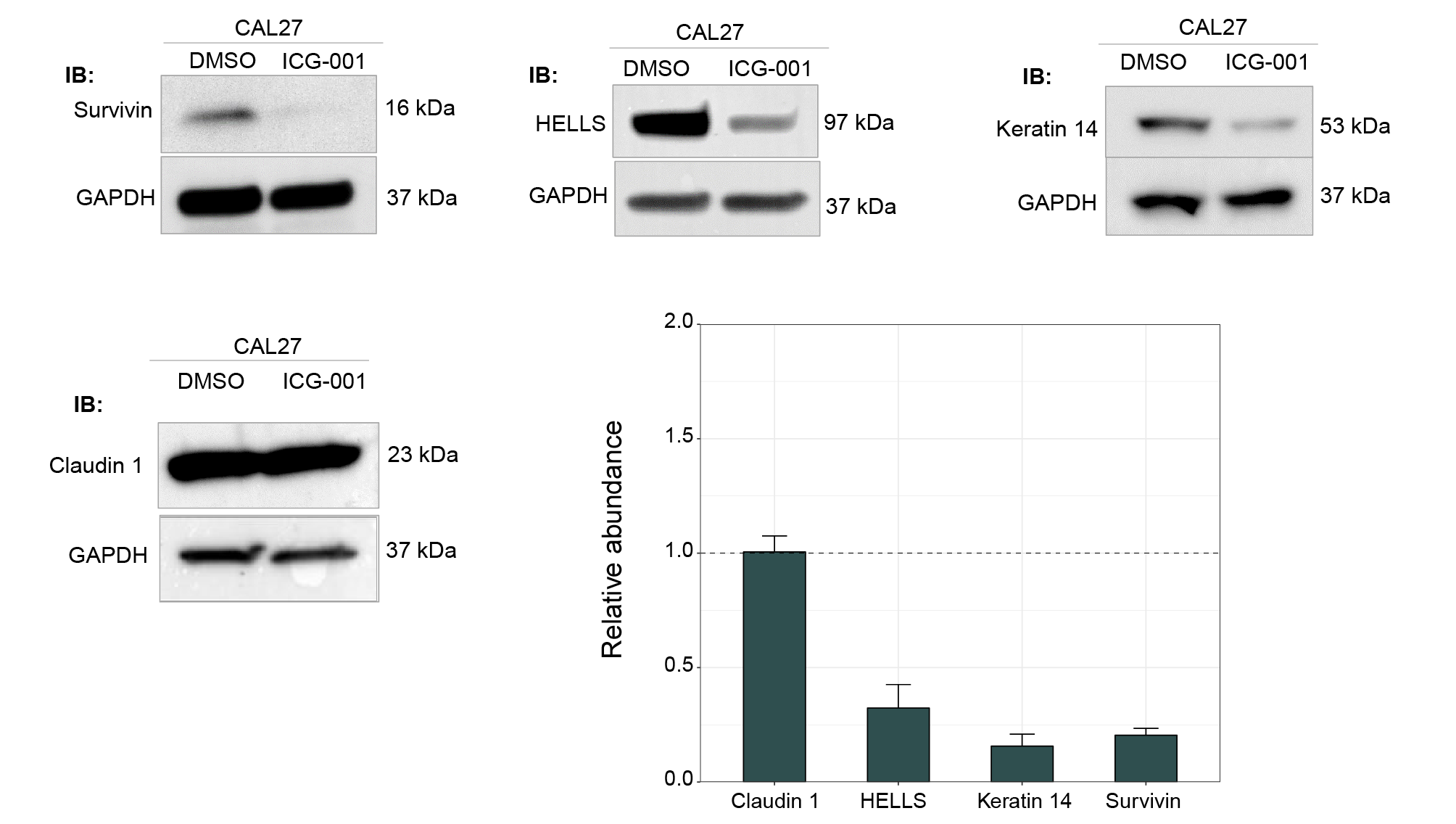


**Figure S4**. ICG-001 treatment alters protein abundances of select markers in CAL27 cells. CAL27 cells were treated with either DMSO (control) or ICG-001, and total cell lysates were then used for immunoblot assay of specific markers (see manuscript Fig. 3). ICG-001 down-regulates HELLS, keratin 14 and survivin proteins. Bar plot of relative abundances for each marker are shown as mean + s.d. values, normalized to DMSO control (dashed line), using GAPDH as a loading control (*n*=2). IB: immunoblot.


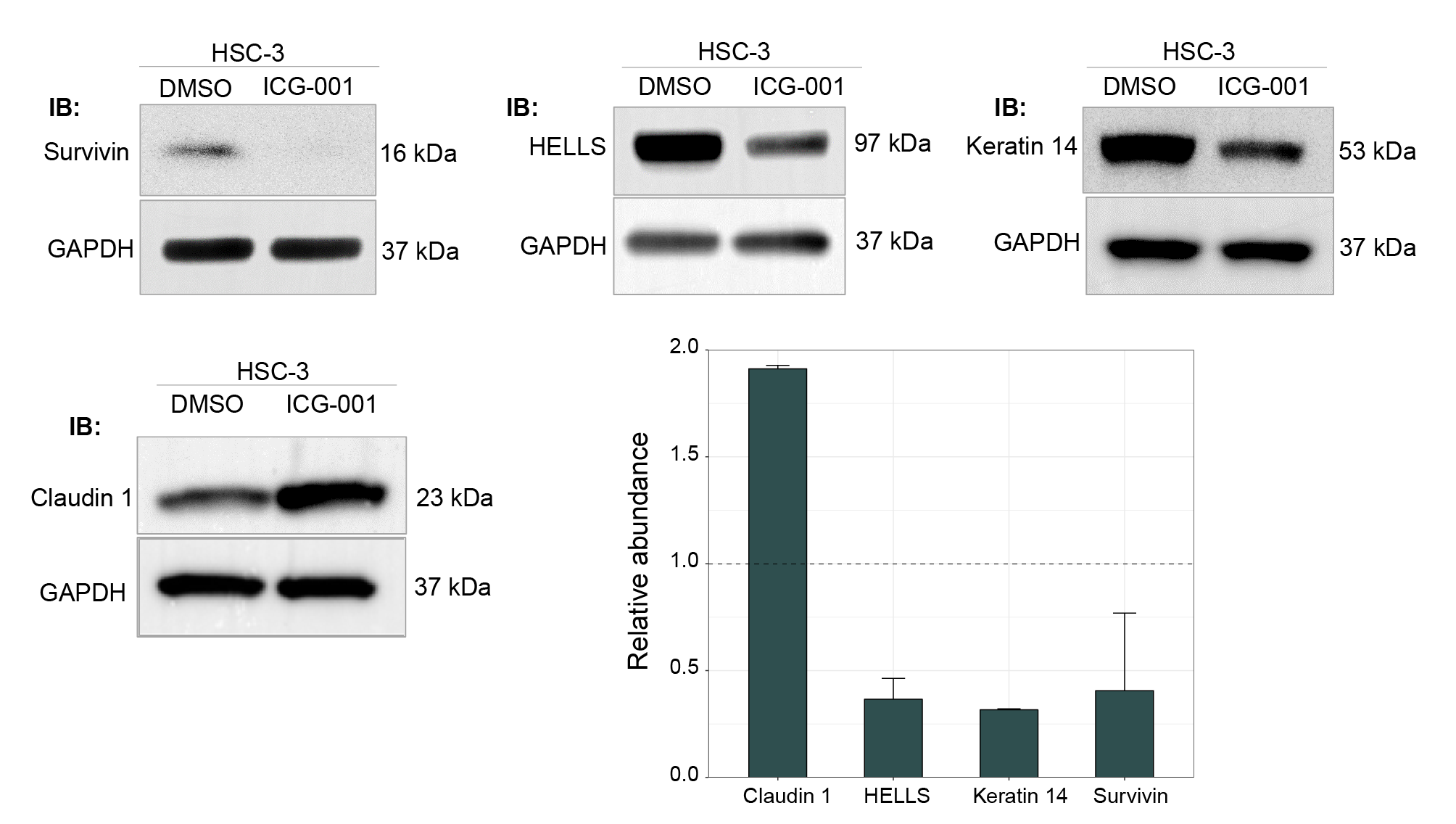


**Figure S5**. ICG-001 treatment alters protein abundances of select markers in HSC-3 cells. HSC-3 cells were treated with either DMSO (control) or ICG-001, and total cell lysates were then used for immunoblot assay of specific markers (see manuscript Fig. 3). ICG-001 down-regulates HELLS, keratin 14 and surviving, while up-regulating differentiation marker claudin 1. Bar plot of relative abundances for each marker are shown as mean + s.d. values, normalized to DMSO control (dashed line), using GAPDH as a loading control (*n*=2). IB: immunoblot.


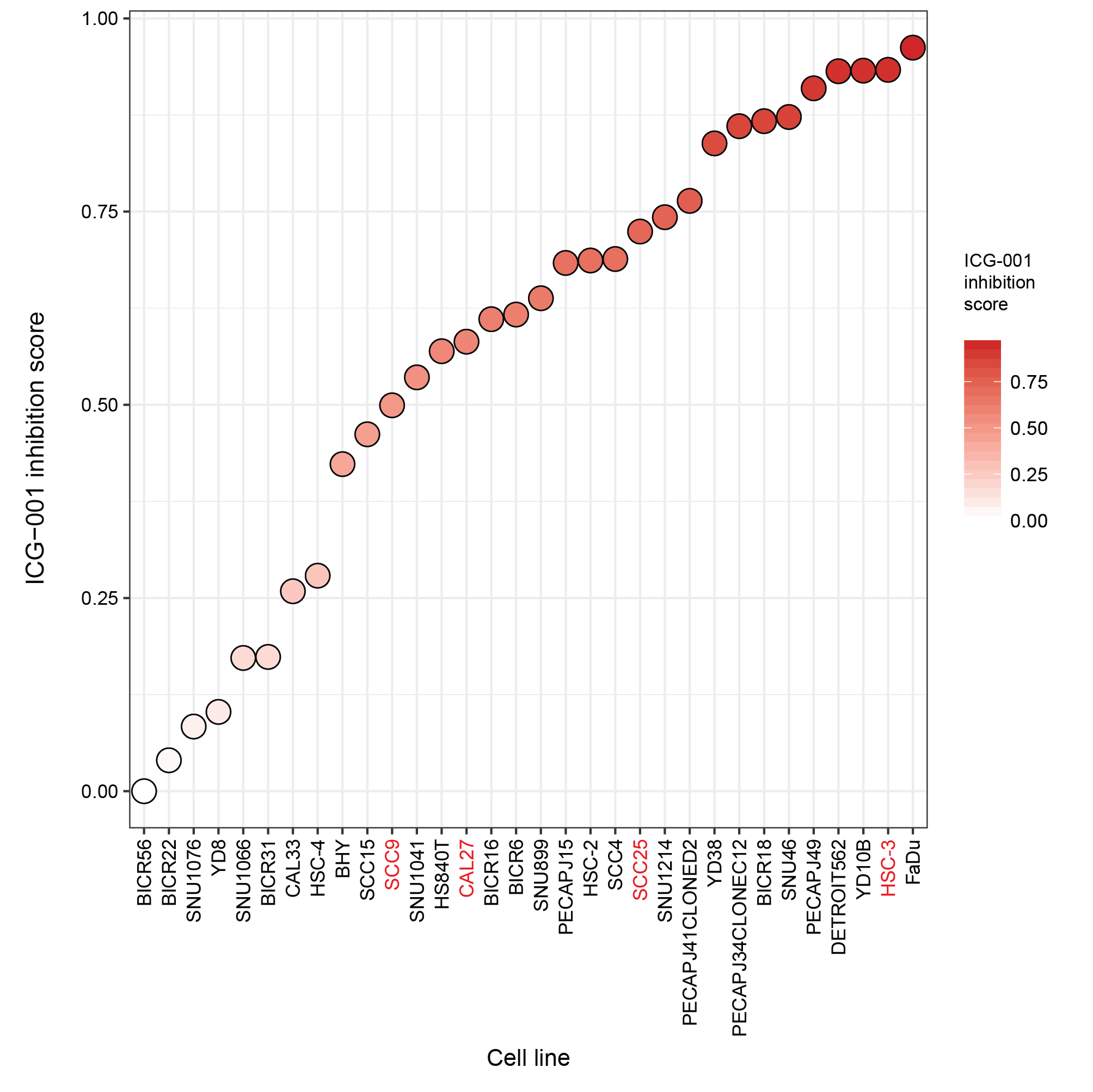


**Figure S6**. Core ICG-001 inhibition gene signature stratifies HNSCC cell lines based on ICG-001 sensitivity estimates. Microarray data pertaining to cells lines originating in the upper aero-digestive tract (UAD) from the cancer cell line encyclopedia (CCLE; *n*=32) was projected in the space of the set of genes significantly down-regulated in both CAL27 and HSC-3 cells (*n*=104; Fig. 2b) using the ASSIGN algorithm, yielding estimates based on the coordinated expression of the gene set (referred to as the ICG-001 inhibition score) for each cell line. Cell lines were then ranked in increasing order of ICG-001 inhibition score estimates. HSC-3 cells are ranked second with relatively higher ICG-001 inhibition scores compared to the other three HNSCC cell lines assessed in this study (SCC25, CAL27 and SCC9; red labels), with similar ranking based on their EC_50_ measurements (Fig. 1). FaDu cells show similar ICG-001 inhibition scores relative to HSC-3, with the highest ranking overall, and were chosen for further characterization (Fig. S7 below).


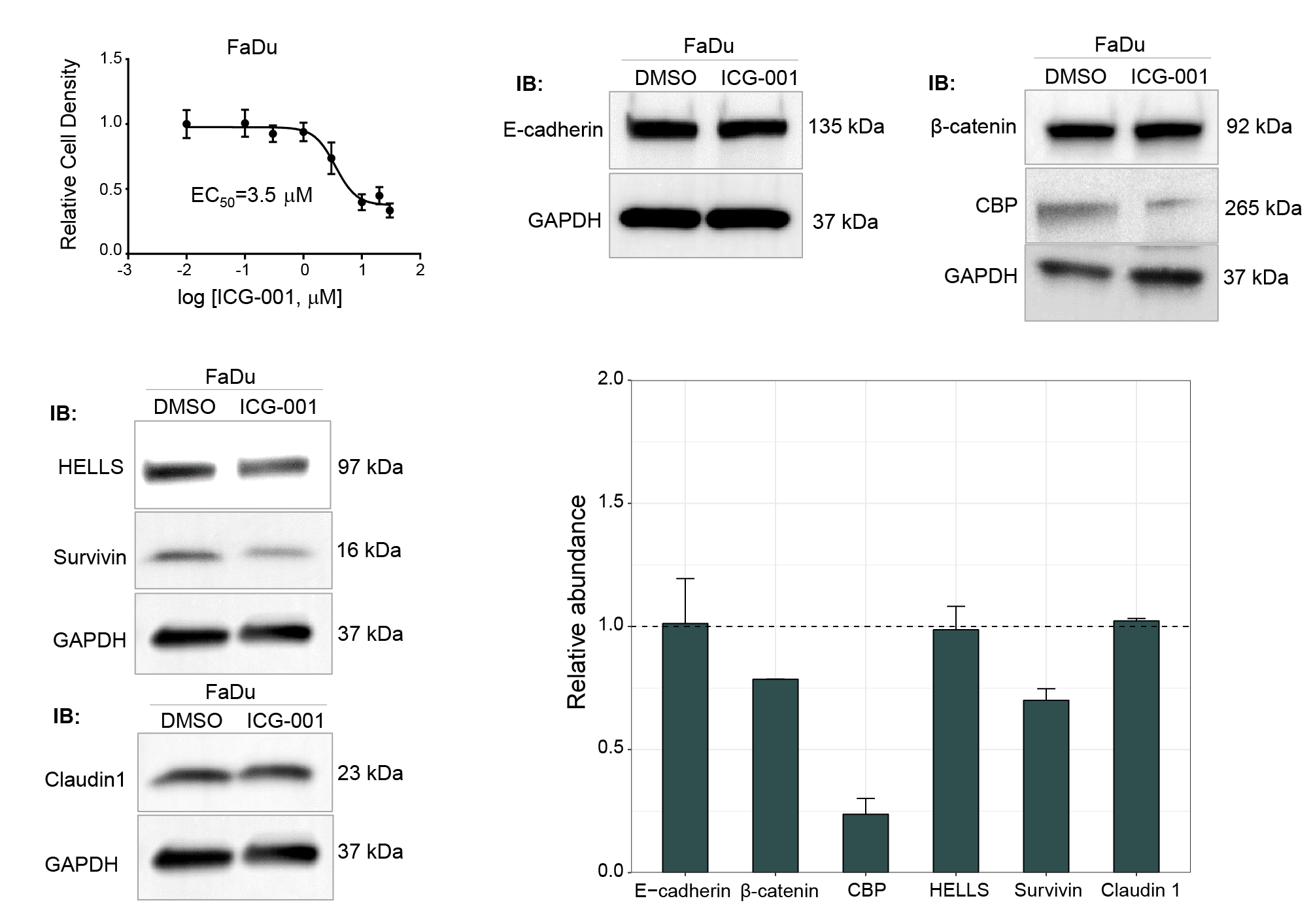


**Figure S7**. Determination of EC_50_ concentration and protein marker quantification of FaDu cells treated with ICG-001. EC_50_ of FaDu cells for ICG-001 treatment was determined using IncuCyte live-cell imaging as described for other HNSCC cell lines (see manuscript Methods). FaDu cells show sensitivity to ICG-001 treatment, as determined by the graded dose response curve, with EC_50_ levels falling between HSC-3, SCC25, and SCC9, CAL27 cells, respectively. Immunoblot assays of FaDu cells treated with either ICG-001 or DMSO control show that CBP and survivin proteins are down-regulated upon ICG-001 treatment, with other markers not altered. Bar plot of relative abundances for each marker are shown as mean + s.d. values, normalized to DMSO control (dashed line), using GAPDH as a loading control (*n*=2 for all markers except β-catenin). IB: immunoblot.

**
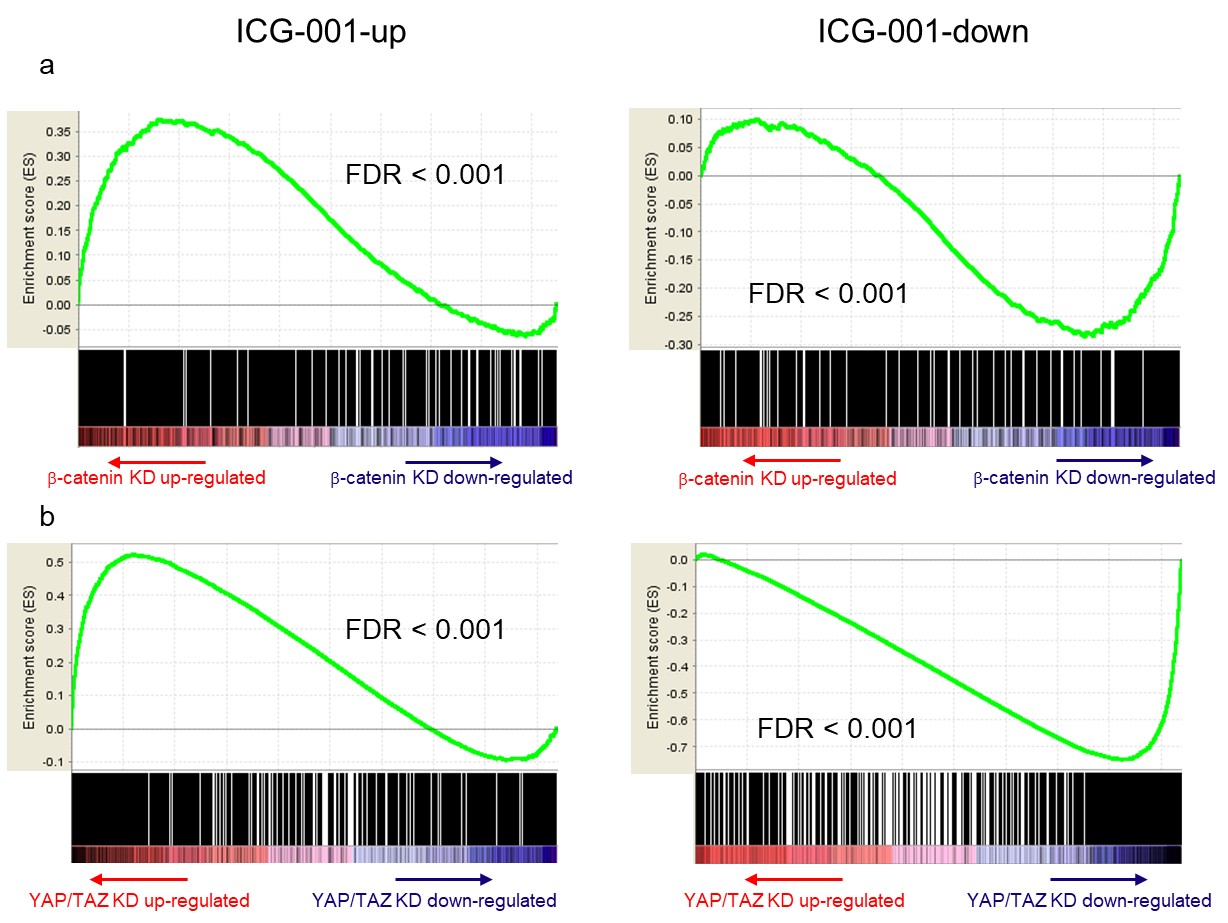
**

**Figure S8.** GSEA of *in vitro* treatment signatures indicates ICG-001 treatment targets β-catenin- and YAP/TAZ-mediated transcriptional activity in OSCC. siRNA-mediated knockdown (KD) of β-catenin was performed in HSC-3 cells, followed by gene expression profiling using microarrays. Additionally, previously generated transcriptional profiling data for YAP/TAZ KD in HSC-3 cells was used [5]. A ranked reference list of genes was generated in decreasing order of *t*-statistic for differential expression, obtained by comparing the KD (*n*=3) versus scrambled siRNA control (*n*=3) groups (*n*=23,744 and 23,466 genes for β-catenin and YAP/TAZ KD experiments, respectively). The ICG-001 treatment signature (both up- and down-regulated genes) were queried for enrichment with respect to each of the KD ranked reference gene lists. Mountain plots for GSEA with respect to β-catenin (**a**) and YAP/TAZ (**b**) KD indicate ICG-001-up- and down-regulated genes are significantly skewed towards up- and down-regulation upon β-catenin or YAP/TAZ KD, respectively. Reported FDR q-values were computed as part of the GSEA software.

# References

1. Veeman MT, Slusarski DC, Kaykas A, Louie SH, Moon RT. Zebrafish prickle, a modulator of noncanonical Wnt/Fz signaling, regulates gastrulation movements. Curr. Biol. 2003;13:680–5.

2. Teo J-L, Ma H, Nguyen C, Lam C, Kahn M. Specific inhibition of CBP/beta-catenin interaction rescues defects in neuronal differentiation caused by a presenilin-1 mutation. Proc. Natl. Acad. Sci. U. S. A. [Internet]. 2005;102:12171–6. Available from: http://www.pubmedcentral.nih.gov/articlerender.fcgi?artid=1189325&tool=pmcentrez&rendertype=abstract

3. Liu G, Sengupta PK, Jamal B, Yang HY, Bouchie MP, Lindner V, et al. N-glycosylation induces the CTHRC1 protein and drives oral cancer cell migration. J. Biol. Chem. 2013;288:20217–27.

4. Subramanian A, Tamayo P, Mootha VK, Mukherjee S, Ebert BL, Gillette M a, et al. Gene set enrichment analysis: a knowledge-based approach for interpreting genome-wide expression profiles. Proc. Natl. Acad. Sci. U. S. A. [Internet]. 2005;102:15545–50. Available from: http://www.ncbi.nlm.nih.gov/pubmed/16199517

5. Hiemer SE, Zhang L, Kartha VK, Packer TS, Almershed M, Noonan V, et al. A YAP/TAZ-Regulated Molecular Signature Is Associated with Oral Squamous Cell Carcinoma. Mol. Cancer Res. [Internet]. 2015 [cited 2015 Jul 1];13:957–68. Available from: http://mcr.aacrjournals.org/content/13/6/957.long
